# Gene regulation, alternative splicing, and posttranslational modification of troponin subunits in cardiac development and adaptation: a focused review

**DOI:** 10.3389/fphys.2014.00165

**Published:** 2014-04-30

**Authors:** Juan-Juan Sheng, Jian-Ping Jin

**Affiliations:** Department of Physiology, Wayne State University School of MedicineDetroit, MI, USA

**Keywords:** isoforms, cardiac muscle, myofilament proteins, posttranslational modification, cardiomyopathy

## Abstract

Troponin plays a central role in regulating the contraction and relaxation of vertebrate striated muscles. This review focuses on the isoform gene regulation, alternative RNA splicing, and posttranslational modifications of troponin subunits in cardiac development and adaptation. Transcriptional and posttranscriptional regulations such as phosphorylation and proteolysis modifications, and structure-function relationships of troponin subunit proteins are summarized. The physiological and pathophysiological significances are discussed for impacts on cardiac muscle contractility, heart function, and adaptations in health and diseases.

The primary contractile unit of striated muscles, e.g., the vertebrate cardiac and skeletal muscles, is the sarcomere. A sarcomere is comprised of overlapping myosin thick filaments and actin thin filaments. The interaction between myosin and actin activates myosin ATPase and powers myofilament sliding and muscle contraction. This process is regulated by the level of cytosolic Ca^2+^ through the thin filament-associated troponin-tropomyosin system (Gordon et al., 2000).

Troponin plays a central role in regulating the contraction and relaxation of striated muscles. The structure and function of troponin have been extensively investigated in the past four decades as comprehensively summarized in several recent review articles (Murphy, 2006; Jin et al., 2008; Wei and Jin, 2011). To provide an overview of the current understanding of the function and regulation of troponin in cardiac muscle, the present review focuses on the isoform genes, splice-forms and posttranslational modifications of troponin in cardiac function during postnatal development and physiological and pathophysiological adaptations.

## The three subunits of troponin complex in vertebrate striated muscle

**Figure 1:**
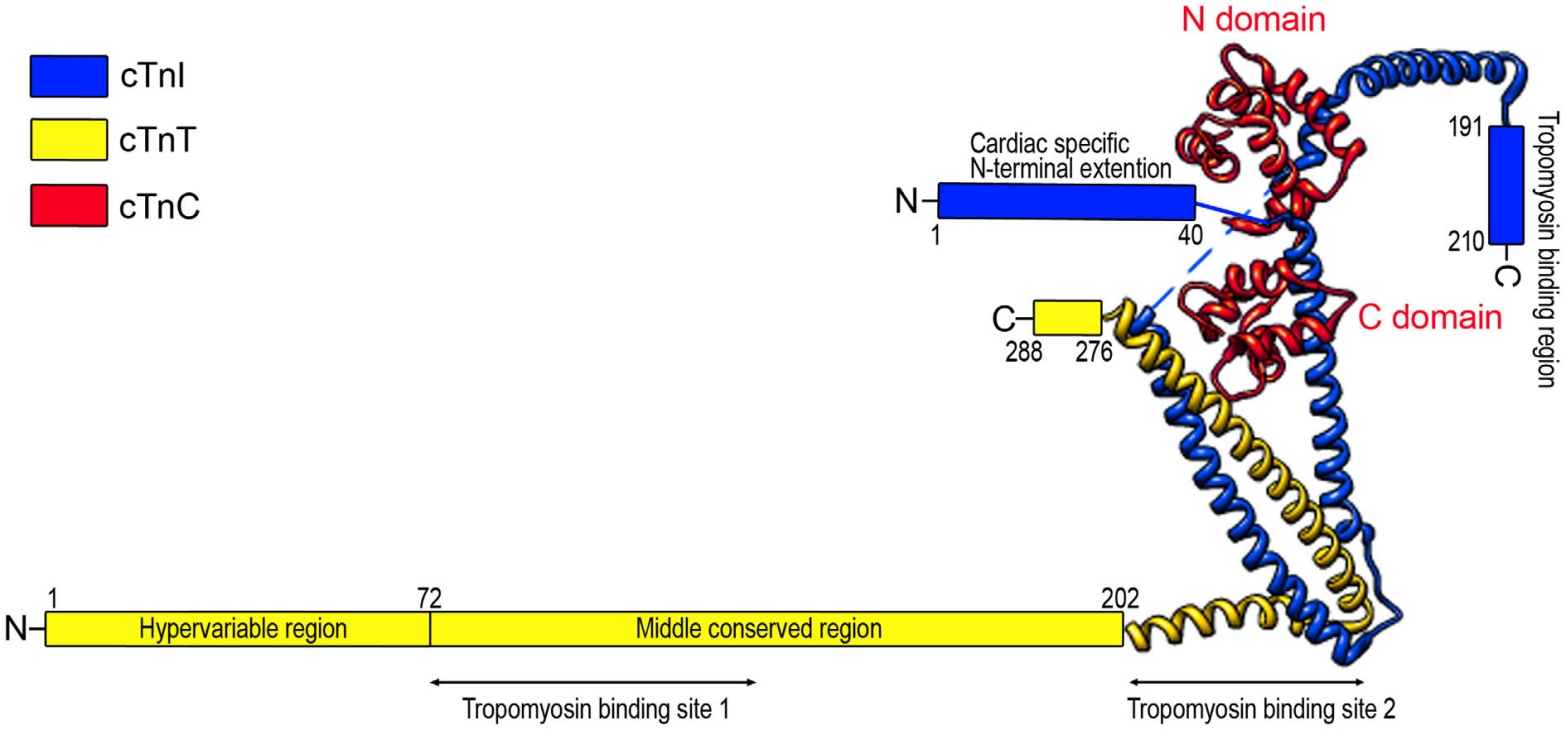
**Schematic structure of cardiac troponin**. Troponin is a protein complex consisting of three subunits: TnC, TnI, and TnT. The structure of troponin can be divided into two structural domains: the core domain and the TnT extension. High resolution crystallographic structure (Takeda et al., 2003) further revealed that the core domain contains two structural subdomains: the regulatory head (residues 3–84 of TnC and residues 150–159 of TnI) and the I-T arm (residues 93–161 of TnC, residues 42–136 of TnI, and residues 203–271 of TnT). The crystal structure portion of the illustration was redrawn from Takeda et al. (2003) using UCSF (Chimera software, alpha version 1.3).

The troponin complex is a heterotrimer consisting of three protein subunits. Named according to their functions, they are the Ca^2+^-binding subunit troponin C (TnC), the actomyosin ATPase inhibitory subunit troponin I (TnI), and the tropomyosin-binding subunit troponin T (TnT) (Greaser and Gergely, 1971) (Figure 1). Low-resolution X-ray crystallography (White et al., 1987) and electron microscopic (Flicker et al., 1982) studies demonstrated that the troponin complex may be divided into two structural domains: The TnT extension that binds tropomyosin and the core domain that bears most of the regulatory function of troponin. High-resolution crystallographic structure further revealed that the core domain of cardiac troponin contains two structurally distinct subdomains that are the regulatory head (amino acid residues 3–84 of TnC and amino acid residues 150–159 of TnI) and the I-T arm (residues 93–161 of TnC, residues 42–136 of TnI and residues 203–271 of TnT). They are dominated with α-helices connected by flexible linkers that make the molecule asymmetric and highly flexible, a crucial feature for the function of troponin in the regulation of muscle contraction and relaxation (Takeda et al., 2003; Vinogradova et al., 2005).

The three troponin subunits are encoded by separate genes. Each of the genes had evolved into muscle type-specific isoform genes. Their expression is regulated during embryonic and postnatal development as well as physiological and pathological adaptations (Jin et al., 2008; Chong and Jin, 2009; Wei and Jin, 2011). TnI and TnT both have three muscle type-specific isoforms encoded by slow skeletal muscle TnI (*TNNI1*), fast skeletal muscle TnI (*TNNI2*), cardiac TnI (*TNNI3*), slow skeletal muscle TnT (*TNNT1*), fast skeletal muscle TnT (*TNNT3*), and cardiac TnT (*TNNT2*) genes. These TnI and TnT isoform genes are closely linked in three tandem pairs in the vertebrate genomes: Fast TnI-fast TnT (*TNNI2-TNNT3*), cardiac TnI-slow TnT (*TNNI3-TNNT1*) and slow TnI-cardiac TnT (*TNNI1-TNNT2*) (Jin et al., 2008; Chong and Jin, 2009; Feng et al., 2009b), supporting the hypothesis that TnI and TnT genes were duplicated from one common ancestral gene.

In contrast to the presence of three muscle type-specific TnI and TnT isoform genes, TnC is present in only two isoforms in the three striated muscle fiber types. Whereas fast skeletal muscle expresses fast TnC encoded by *TNNC2*, mature cardiac muscle and slow skeletal muscle share one isoform, the cardiac/slow skeletal muscle TnC encoded by *TNNC1* (Schreier et al., 1990; Collins, 1991; Prigozy et al., 1997).

The diversity of isoform genes encoding the subunits of troponin endues the heart with adaptation during development. Discussed in more details in later sections, embryonic heart expresses solely slow TnI paired with cardiac TnT. An isoform transition from slow TnI to solely cardiac TnI in adult heart occurs during development. Cardiac TnI has a heart-specific N-terminal extension that is a regulatory structure specific to the adult cardiac muscle (Chong and Jin, 2009). On the other hand, slow TnI expression in embryonic hearts increases Ca^2+^ sensitivity of myofilament and the tolerance to acidosis, although it diminishes length dependence of Ca^2+^ activation (Arteaga et al., 2000). Cardiac TnT also has an N-terminal variable region that undergoes developmentally regulated alternative splicing (Jin and Lin, 1988, 1989) whereas no alternative RNA splicing is found for the transcripts of any of the three TnI isoform genes.

## Troponin C

Troponin C belongs to the calmodulin super family of genes, containing four EF-hand helix coil-helix divalent metal ion-binding sites (Collins, 1991; Kawasaki et al., 1998). TnC is a dumbbell-shaped molecule with the N- and C-terminal globular domains connected by a nine turn α-helix (Herzberg and James, 1985). The C-terminal domain of TnC contains two high affinity Ca^2+^ or Mg^2+^ binding sites (Site III and Site IV), which are primarily occupied by Mg^2+^ in resting muscle cells and can become partially bound with Ca^2+^ during the activation of contraction (Robertson et al., 1981). The C-terminal domain of TnC plays a structural role of maintaining the anchoring affinity of the whole troponin complex to the thin filament (Zot and Potter, 1982).

The N-terminal domain of fast skeletal muscle TnC contains two low affinity metal ion-binding sites designated as Site I and Site II that are regulatory Ca^2+^-binding sites responsible for the regulation of muscle contraction (Sheng et al., 1990; Sweeney et al., 1990). The transient rise of cytosolic Ca^2+^ during the activation of contraction results in Ca^2+^ binding to the N-terminal domain of TnC and induces a cascade of conformational changes in the troponin complex and sarcomeric thin filament (Robertson et al., 1981; Collins, 1991; Gordon et al., 2000; Solaro, 2010). The conformational changes increase the binding affinity of TnC for TnI, promoting a detachment of TnI from actin, which releases the inhibition of actomyosin ATPase and activates myofilament sliding and shortening of the sarcomere (Grabarek et al., 1992).

Different from the fast skeletal muscle TnC, the N-terminal domain of cardiac/slow TnC contains only one active Ca^2+^ binding site (Site II), whereas Site I had lost the ability of binding Ca^2+^ (Van Eerd and Takahashi, 1976). Elimination of Ca^2+^ binding Site II in cardiac/slow TnC renders a cardiac fiber insensitive to Ca^2+^, whereas reengineering an active Ca^2+^-binding Site I does not compensate for the effect of Site II mutation. Therefore, Site II plays a critical role responsible for the regulatory function of cardiac/slow TnC (Sweeney et al., 1990). Nonetheless, engineered cardiac/slow TnC in which both Site I and Site II are actively binding to Ca^2+^ showed increased Ca^2+^ sensitivity than that of wild type cardiac TnC in which only Site II is active (Sweeney et al., 1990). The Ca^2+^ sensitivity of cardiac/slow TnC can also be regulated by other myofilament proteins, such as TnI, TnT, tropomyosin, actin, myosin binding protein-C, and myosin (Blumenschein et al., 2001; Burkart et al., 2003a). No alternative splicing or posttranslational regulation of TnC has been observed during development or pathological adaptations.

## Troponin I

Troponin I is the inhibitory subunit of the troponin complex. In the absence of Ca^2+^, its inhibitory region binds with actin, and thereby inhibits actomyosin ATPase (Farah et al., 1994). In the presence of Ca^2+^, the C-terminal domain of TnC interacts with TnI to induce conformational changes of TnI, releases the inhibitory effect, and initiates muscle contraction (Farah et al., 1994; Perry, 1999).

In vertebrate striated muscles, the three TnI isoform genes (Hastings, 1997; Perry, 1999; Chong and Jin, 2009) are differentially expressed under fiber type-specific and developmentally regulated transcriptional control. Fast skeletal muscle fibers only express fast TnI, and slow skeletal muscle fibers express only slow TnI. Accordingly, a slow to fast TnI (and TnT) isoform switching occurs during the slow to fast fiber type transition in muscle adaptation to unloading (Stevens et al., 2002; Yu et al., 2007).

As indicated above, cardiac muscle switches TnI isoforms during development (Saggin et al., 1989). The slow skeletal muscle TnI gene is expressed in the embryonic heart and switches off during development. Around birth, the expression of cardiac TnI gene up-regulates to completely replace slow TnI in adult cardiac muscle (Saggin et al., 1989; Sasse et al., 1993). The adult heart expresses cardiac TnI as the sole isoform and it does not change under pathological conditions such as ischemic heart disease, dilated cardiomyopathy, or end-stage heart failure (Sasse et al., 1993). This developmental TnI isoform transition may contribute to the differences in the Ca^2+^ sensitivity and pH responsiveness of force development of cardiomyocytes (Westfall et al., 1999). Over-expression of slow TnI in cardiac muscle of adult transgenic mice impaired cardiomyocyte relaxation and diastolic cardiac function due to increased Ca^2+^ sensitivity (Fentzke et al., 1999). On the other hand, slow TnI increased the tolerance of cardiomyocytes to acidosis-induced decrease in myofilament Ca^2+^ sensitivity (Westfall et al., 2000). These findings indicate that slow TnI produces a higher Ca^2+^ affinity of the troponin complex than that of cardiac TnI, which may maintain Ca^2+^ sensitivity of myofilament at the lower pH (6.5 vs. 7.0) in embryonic cardiomyocytes (Solaro et al., 1988).

### Structural features of cardiac TnI

Based on *in vitro* structure-function relationship studies, the structure of cardiac TnI can be divided into six functional segments (Li et al., 2004) (Figure 1): (a) cardiac-specific N-terminal extension (amino acids 1–30) that is not present in fast TnI and slow TnI; (b) an N-terminal region (amino acids 42–79) that binds the C domain of TnC; (c) a TnT-binding region (amino acids 80–136); (d) the inhibitory peptide (amino acids 128–147) that interacts with TnC and actin–tropomyosin; (e) the switch or triggering region (amino acids 148–163) that binds the N domain of TnC; and (f) the C-terminal region (amino acids 164–210) that binds actin–tropomyosin and is the most conserved segment highly similar among isoforms and across species (Jin et al., 2001, 2008). Recent studies demonstrated that the last 20 amino acids of the C-terminal end segment of TnI (amino acids 191–210), encoded by exon 8 is a Ca^2+^-modulated allosteric structure (Jin et al., 2001; Zhang et al., 2011a). Protein binding experiments showed that this segment functions through Ca^2+^-regulated conformational changes and interactions with tropomyosin (Solaro et al., 2008; Zhang et al., 2011a).

### Phosphorylation of cardiac TnI

There is no alternative RNA splicing found for the transcripts of any TnI genes. In the meantime, posttranslational modifications have major roles in regulating the structure and function of cardiac TnI (Solaro et al., 2008). The mechanisms include amino acid side chain modifications and cleavages of the polypeptide chain, which induce conformational changes that modify the interaction with cardiac TnC and effects on cardiac muscle contractility (Pi et al., 2003; Layland et al., 2005; Westfall et al., 2005; Solaro and Van Der Velden, 2010; Akhter et al., 2012). Phosphorylation also regulates the degradation of cardiac TnI (Di Lisa et al., 1995).

It is well-accepted that phosphorylation of cardiac TnI at Ser_23_ and Ser_24_ in the adult heart-specific N-terminal extension regulates the diastolic function of cardiac muscle (Solaro and Kobayashi, 2011). Compiling evidences showed that Ser_23_ and Ser_24_ are sequentially phosphorylated by protein kinase A (PKA) under the regulation of adrenergic signaling cascades (Quirk et al., 1995; Solaro et al., 2008; Solaro and Kobayashi, 2011; Rao et al., 2012), reducing the Ca^2+^-binding affinity of the N domain regulatory site of cardiac TnC (Zhang et al., 1995b) and enhancing diastolic function of cardiac muscle (Zhang et al., 1995a; Stelzer et al., 2007; Li et al., 2010). Bisphosphorylation at Ser_23_ and Ser_24_ results in weakening interactions of cardiac TnI with the N-lobe of cardiac TnC and favoring the intra-molecular interaction between the N-terminal extension and the inhibitory region of cardiac TnI (Howarth et al., 2007). These two serine residues have also been reported to be phosphorylated *in vitro* by PKC-β, PKC-ε (Kobayashi et al., 2005), PKD (previously named PKCμ) (Haworth et al., 2004; Cuello et al., 2007; Bardswell et al., 2010) and PKG (Layland et al., 2002).

While PKA phosphorylation of cardiac TnI at Ser_23_/Ser_24_ increases myocardial relaxation, PKC phosphorylation of cardiac TnI exerts an antithetic role (Sakthivel et al., 2005; Kooij et al., 2011). PKC phosphorylates cardiac TnI at Ser_43_ and Ser_45_ (residue # in mouse sequence) in the region binding the C domain of TnC and Thr_144_ in the inhibitory region, slowing cardiac relaxation and increasing the duration of calcium transient and twitch contraction (Macgowan et al., 2001; Pi et al., 2002; Burkart et al., 2003b; Westfall et al., 2005). In mouse heart, phosphorylation of Thr_144_ of cardiac TnI by PKC-βII increased myofilament Ca^2+^ sensitivity (Wang et al., 2006). Substitution with Pro at Thr_144_ delayed relaxation, suggesting a role of Thr_144_ in accelerating relaxation in cardiomyocytes (Westfall et al., 2005). However, another study found that Thr_144_ phosphorylation did not modify the thin filament Ca^2+^ sensitivity, but depressed cooperative activation of thin filaments (Lu et al., 2010). The mechanism how phosphorylation of Thr_144_ regulates cardiac troponin requires further investigation.

It was reported that phosphorylation of cardiac TnI at Thr_143_ by PKC impaired the interaction between the inhibitory region and TnC, leading to depressed actomyosin ATPase activity and contractility (Lindhout et al., 2002; Li et al., 2003). PKC phosphorylation of cardiac TnI also inhibited ATPase activity (Noland and Kuo, 1991) and thin filament sliding velocity, which may protect the heart from ischemia-reperfusion injury (Macgowan et al., 2001).

Ser_150_ has also been found to be a phosphorylation site in cardiac TnI, which can be phosphorylated by P21-activated kinase (Pak) to increase the Ca^2+^ sensitivity of cardiac myofibrils (Buscemi et al., 2002; Ke et al., 2004). Recently, it was demonstrated that AMP-activated Protein Kinase (AMPK) phosphorylates cardiac TnI *in vitro* at Ser_150_ (Oliveira et al., 2012) adjacent to the inhibitory loop (Sancho Solis et al., 2011), which increased sensitivity of calcium-dependent force development (Nixon et al., 2012), prolonged relaxation (Oliveira et al., 2012), and increased the effect of adrenergic-induced myocardial hypertrophy (Taglieri et al., 2011). As AMPK is thought to act as a cellular energy sensor, phosphorylation of Ser_150_ may provide an adaptive mechanism in energy deprivation.

*In vitro* studies showed that human cardiac TnI was phosphorylated by mammalian sterile 20-like kinase 1 (Mst1) at Thr_31_, Thr_51_, Thr_129_, and Thr_143_, among which Thr_31_ is a preferential site (You et al., 2009). Several new phosphorylation sites in the N-terminal region (Ser_5_/Ser_6_/Tyr_26_) have also been identified with decreased phosphorylation in heart failure, whereas phosphorylation of Ser_166_/Thr_181_/Ser_199_ in the C-terminal region and Ser_77_/Thr_78_ at the TnI-TnT interface (I-T arm) was increased (Zhang et al., 2012).

In human end-stage dilated cardiomyopathy, baseline phosphorylation of cardiac TnI was diminished with increased myofilament Ca^2+^ affinity (Zakhary et al., 1999). In failing human heart, the PKA sites Ser_23_/Ser_24_ in cardiac TnI are dephosphorylated (Bodor et al., 1997) and the PKC site Ser_43_/Ser_45_/Thr_144_ are increasingly phosphorylated (Zhang et al., 2012), resulting in ventricular diastolic dysfunction. Cardiac TnI R21C mutation in transgenic mouse heart showed dephosphorylation of Ser_23_/Ser_24_ and developed cardiac hypertrophy and fibrosis (Wang et al., 2012b). In remodeling myocardium after myocardial infarction, expression of PKA was significantly down-regulated in cardiomyocytes and thus PKA-mediated phosphorylation of cardiac TnI was consequently decreased (Van Der Velden et al., 2004). Dephosphorylation of Ser_23_/Ser_24_ in cardiac TnI could also account for the contractile defect in end-stage heart failure (Messer et al., 2007), and the significantly reduced inotropic responsiveness to β-adrenergic stimulation in decompensated cardiac hypertrophy (McConnell et al., 1998).

**Figure 2:**
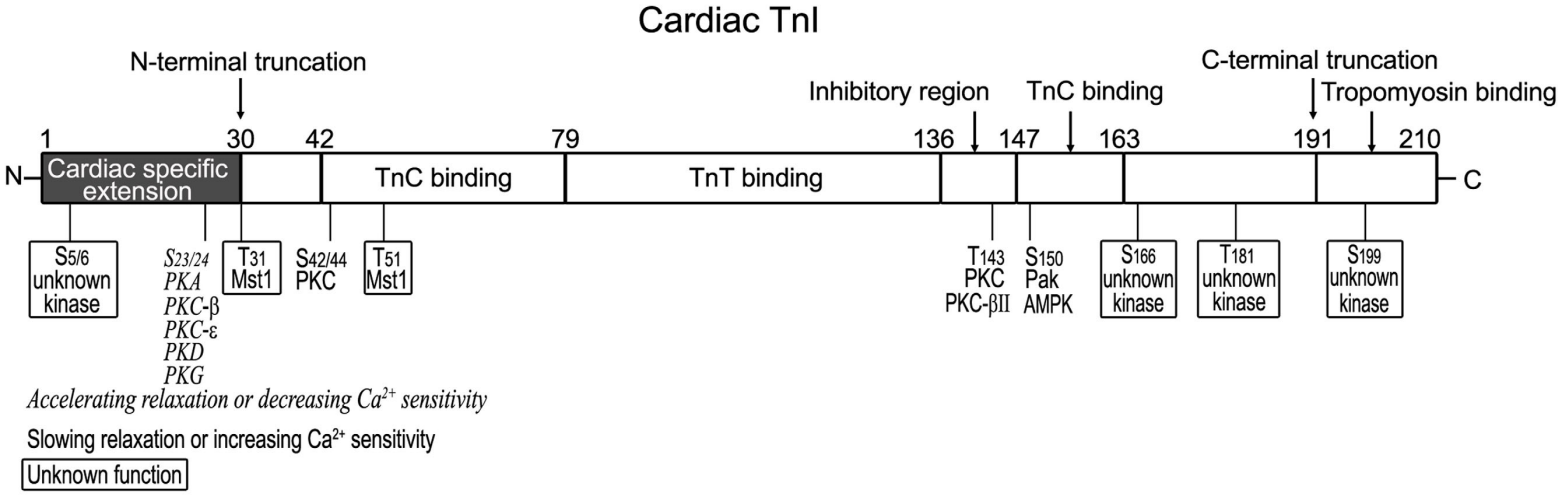
**Structural and functional domains of cardiac TnI and posttranslational modifications**. Indicated on this linear map of cardiac TnI polypeptide (residue # corresponds to that in human sequence including Met1), Ser_23_/Ser_24_ are phosphorylated by PKA (Solaro and Kobayashi, 2011), decreasing Ca^2+^ sensitivity and accelerating relaxation. They have also been reported to be phosphorylated by PKC-β, PKC-ε (Kobayashi et al., 2005), PKD (Haworth et al., 2004; Cuello et al., 2007; Bardswell et al., 2010) and PKG (Layland et al., 2002). Ser_42_/Ser_44_, Thr_143_, and Ser_150_ are phosphorylated by PKC, Pak or AMPK, decreasing Ca^2+^ sensitivity and slowing relaxation (Macgowan et al., 2001; Buscemi et al., 2002; Pi et al., 2002; Burkart et al., 2003b; Westfall et al., 2005). Thr_31_ and Thr_51_ are phosphorylated by Mst1 (You et al., 2009) with unknown function. New phosphorylation sites have been identified in the N-terminal region (Ser_5_/Ser_6_, with decreased levels in heart failure) and in the C-terminal region (Ser_166_/Thr_181_/Ser_199_, with unknown kinases and functions) (Zhang et al., 2012). A restrictive N-terminal truncation of cardiac TnI occurs in adaptation to hemodynamic stresses to selectively remove the adult heart-specific N-terminal extension with an effect on increasing myocardial relaxation, similar to the effect of PKA phosphorylation at Ser_23_/Ser_24_ (Barbato et al., 2005). A deletion of the C-terminal 19 amino acids was found in myocardial ischemia-reperfusion injury (McDonough et al., 1999) and myocardial stunning (McDonough et al., 2001), slowing down the rates of force development and relaxation (Narolska et al., 2006).

The structural-functional domains of cardiac TnI, phosphorylation sites, and proteolytic modifications (see below) are summarized in Figure 2 (all residue #s reflected that in human cardiac TnI and included Met1).

### Proteolytic modifications of cardiac TnI

The half-life of cardiac TnI in adult cardiomyocytes is estimated to be ~3.2 days and there is a pool of unassembled cardiac TnI in the cytoplasm (Martin, 1981), indicating that cardiac TnI is synthesized in excess. Study on transgenic mouse hearts over-expressing modified cardiac TnI demonstrated that the stoichiometry of total TnI is determined by the incorporation into myofilaments (Feng et al., 2009a).

Cardiac TnI is also a substrate of intracellular modifying proteases, with a demonstrated sensitivity to μ-calpain (Di Lisa et al., 1995). Its degradation by μ-calpain was modulated by phosphorylation, in which phosphorylation by PKA reduced the sensitivity of cardiac TnI whereas phosphorylation by PKC increased the sensitivity of cardiac TnI to μ-calpain (Di Lisa et al., 1995).

#### C-terminal truncation

The C-terminal end segment is the most conserved region of the TnI polypeptide (Jin et al., 2001). As an allosteric structure regulated by Ca^2+^ (Jin et al., 2001; Zhang et al., 2011a), it binds and stabilizes tropomyosin in the absence of Ca^2+^ (Galiñska et al., 2010; Zhang et al., 2011a). Mutations R193H and R205H in the C-terminal end segment altered conformation and function of the I-T interface and increased cardiac TnI binding affinity for TnT, indicating the regulatory role of the C-terminal end segment (Akhter et al., 2014).

A deletion of the C-terminal 19 amino acids was found during myocardial ischemia-reperfusion injury in Langendorff perfused rat hearts (McDonough et al., 1999). It was also seen in myocardial stunning in coronary bypass patients (McDonough et al., 2001). Over-expression of the C-terminal truncated cardiac TnI (cTnI_1−192_) in transgenic mouse heart resulted in a phenotype of myocardial stunning, and systolic and diastolic dysfunctions (Murphy et al., 2000). 50% replacement of intact cardiac TnI with cTnT_1−192_ in myofibrils *in vitro* and cardiomyocytes *ex vivo* did not affect maximal tension development but slowed down the rates of force redevelopment as well as relaxation (Narolska et al., 2006). cTnI_1−192_ significantly increased Ca^2+^-activated actomyosin ATPase and sliding velocity as compared with troponin containing intact cardiac TnI (Foster et al., 2003).

However, the pathological significance of the C-terminal truncation of cardiac TnI remains controversial. No C-terminal truncated cardiac TnI was found in swine hearts subjected to *in vivo* regional ischemia-reperfusion (Thomas et al., 1999). Another study suggested that the myocardial stunning in pigs induced by regional ischemia was due to dephosphorylation of phospholamban without degradation of cardiac TnI (Kim et al., 2001). No significant degradation of cardiac TnI was detected in the hearts of conscious dogs after reversible ischemia (Lüss et al., 2000; Sherman et al., 2000). A hypothesis is that the marked elevation of preload after global ischemia in Langendorff perfused heart (>30 mmHg) rather than ischemia *per se* activated μ-calpain and caused cardiac TnI proteolysis (Feng et al., 2001).

#### Restrictive N-terminal truncation

Different from the C-terminal truncation, a selective removal of the N-terminal extension of cardiac TnI has been found to be a regulatory mechanism in cardiac adaptation in physiological and pathological stress conditions. The N-terminal extension of approximately 30 amino acids is an adult heart-specific structure absent in fast and slow skeletal muscle TnI (Perry, 1999; Chong and Jin, 2009). The N-terminal extension contains the PKA phosphorylation sites and plays a role in modulating the overall molecular conformation and function of cardiac TnI (Akhter et al., 2012). A restrictive N-terminal truncation of cardiac TnI occurs at low levels in normal hearts of many species examined including human and significantly increases in adaptation to hemodynamic changes such as that in a tail suspension rat model of simulated microgravity (Yu et al., 2001) and G_sα_ deficiency-caused failing mouse hearts (Feng et al., 2008b).

Experimental evidence showed that the N-terminal extension truncated cardiac TnI (cTnI-ND) increased myocardial relaxation and improved ventricular filling, similar to the effect of PKA phosphorylation (Barbato et al., 2005). While expression of a similarly N-terminal truncated cardiac TnI did not cause functional defect in cardiomyocytes (Guo et al., 1994), over-expression of cTnI-ND improved the diastolic function of *ex vivo* working hearts of G_sα_ deficiency mice (Feng et al., 2008b) and cardiac function *in vivo* in aging mice (Biesiadecki et al., 2010). Co-expression of cTnI-ND corrected the diastolic dysfunction of restrictive cardiomyopathy hearts caused by cTnI^193His^ mutation (Li et al., 2010). Isolated cardiomyocytes from cTnI-ND mouse hearts showed larger shortening amplitude and higher systolic and diastolic velocities (Wei and Jin, 2013). Whereas the N-terminal extension of cardiac TnI does not directly interact with other known proteins in the thin filament regulatory system, the molecular mechanism of cTnI-ND's function involves alterations of the conformation and function of the middle region of cardiac TnI (Akhter et al., 2012).

A study on trout cardiac TnI that lacks the N-terminal extension showed that troponin complex containing trout cardiac TnI had a greater Ca^2+^ affinity than human troponin (Kirkpatrick et al., 2011). Although trout cardiac TnI lacks the two substrate residues of PKA phosphorylation, myofilament Ca^2+^ affinity decreased when treated with PKA, similar to the response of mammalian cardiac TnI with the N-terminal extension (Kirkpatrick et al., 2011). This apparently N-terminal extension-independent PKA regulation and enhancement of relaxation is worth further investigation.

## Troponin T

Troponin T is a striated muscle-specific protein of ~250–305 amino acids with molecular weights ranging from 31-kDa to 36-kDa. Same as the differentiated TnI isoform genes, three muscle type-specific TnT isoform genes are present in vertebrates and expressed in fiber-specific and developmentally regulated manner (Jin et al., 2008; Wei and Jin, 2011). In addition to specific expression in cardiomyocytes, cardiac TnT also expresses at significant levels in embryonic skeletal muscle (Anderson et al., 1991; Jin, 1996) and myopathic skeletal muscle of patients and Duchenne muscular dystrophy (Ricchiuti and Apple, 1999), likely indicating active growth or regeneration.

### Structural and functional domains of TnT

**Figure 3:**
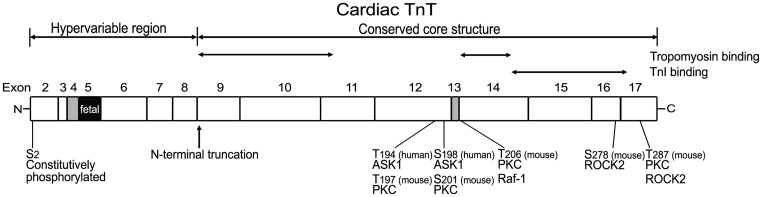
**Structural and functional domains of cardiac TnT, alternative spliced exons, and posttranslational modifications**. Outlined on this linear map of cardiac TnT polypeptide (residue #s are those published in the original papers, which used various isoforms from different species), the functional segments T1, T2, and the N-terminal hypervariable region as well as the alternatively spliced exons 4, 5, and 13 are indicated. Ser_2_ is a highly conserved residue constitutively and phosphorylated in cardiac TnT *in vivo* (Perry, 1998; Sancho Solis et al., 2008). *In vitro* studies demonstrated that cardiac TnT could be phosphorylated by PKC at Thr_197_, Ser_201_, Thr_206_, and Thr_287_ in the C-terminal region that contains binding sites for TnI, TnC, and tropomyosin (Jideama et al., 1996). Thr_206_ can also be phosphorylated by Raf-1 (Pfleiderer et al., 2009) and Ser_278_ and Thr_287_ by ROCK2, which inhibited tension development and ATPase activity in skinned fibers (Vahebi et al., 2005). Thr_194_ and Ser_198_ of cardiac TnT have been found to be phosphorylated by ASK1 with decreases in cardiomyocyte contractility (He et al., 2003).

Earlier studies had dissected the structure of TnT into two functional regions based on fragmentation using limited cleavages with chymotrypsin and CNBr, i.e., the T1 and T2 fragments (amino acids1–158 and 159–259, respectively, in rabbit skeletal muscle TnT) (Tanokura et al., 1983; Perry, 1998) (Figure 3). The T1 fragment binds the head-tail junction of tropomyosin mainly through a 39 amino acids segment in the N-terminal portion of the conserved middle region of TnT (Jin and Chong, 2010). The C-terminal T2 fragment contains binding sites for TnC, TnI, and F-actin (Heeley et al., 1987; Schaertl et al., 1995; Perry, 1998) as well as another tropomyosin-binding site in a segment of 25 amino acids near the beginning of the T2 region (Jin and Chong, 2010), which binds the central region of tropomyosin (Morris and Lehrer, 1984) (Figure 3). The current model states that TnT plays an anchoring role and transmits the signal from Ca^2+^-TnC binding to the thin filament regulatory system in striated muscles (Perry, 1998).

The N-terminal region of TnT is a “hypervariable” region (Perry, 1998). This region has variable lengths and variable amino acid sequences. Cardiac TnT is of larger size than fast and slow skeletal muscle TnT, mainly due to a longer N-terminal variable region (Perry, 1998; Wei and Jin, 2011). The N-terminal variable region of TnT does not contain any binding sites for other known myofilament proteins (Perry, 1998; Jin et al., 2008; Wei and Jin, 2011).

Taking advantage of the presence of a cluster of transition metal ion-binding sites in the N-terminal variable region of fast skeletal muscle TnT of avian species in the orders of *Galliformes* and *Craciformes* (Jin and Smillie, 1994), antibody epitope analyses showed that Zn^2+^-binding to the N-terminal region of chicken breast muscle fast TnT altered the molecular conformation of epitopes outside of the N-terminal region, demonstrating a long-range modulatory effect (Wang and Jin, 1998). Fluorescence spectral analysis further showed that Cu^2+^ binding to the N-terminal region of chicken fast TnT induced changes in fluorescence intensity and anisotropy of Trp_234_, Trp_236_, and Trp_285_ or fluorescein-labeled Cys_263_ in the C-terminal region (Jin and Root, 2000). Protein-binding studies showed that the binding of Zn^2+^ to the N-terminal region of chicken fast TnT decreased the binding affinity for tropomyosin, TnI, and TnC (Ogut and Jin, 1996; Jin et al., 2000). These data indicated that the N-terminal variable region modulates the conformation and function of TnT core structure to fine-tune muscle contractility (Biesiadecki et al., 2007a).

### Evolutionarily selected utilization of slow skeletal muscle TnT in toad heart

We recently found that the heart of adult toads (*Bufo*) exclusively expresses slow skeletal muscle TnT instead of cardiac TnT while all other myofilament proteins, including cardiac TnI and cardiac myosin, remain to be the normal cardiac isoform (Feng et al., 2012). This unique biochemical content of toad cardiac muscle is correlated to a striking physiological feature of toads, i.e., it is highly adaptive to large changes in the volumes of body fluid and blood between rain and dry seasons (Boral and Deb, 1970) or under experimental conditions (Deb et al., 1974).

Functional studies demonstrated that toad hearts had faster contractile and relaxation velocities and a significantly higher tolerance to afterload (Feng et al., 2012). These findings demonstrate that the selective utilization of slow skeletal muscle TnT in toad heart was an adaptive change with significantly functional advantage and fitness value during evolutionary selection. This observation suggests that altering TnT function may be targeted for the improvement of systolic function and the treatment of congestive heart failure.

No expression of cardiac TnT was detected in either heart or skeletal muscle of the toad (Feng et al., 2012). Despite the unusual expression in the heart, slow skeletal muscle TnT is normally expressed specifically in toad slow twitch skeletal muscles (Feng et al., 2012). The mechanism of selectively activating the slow skeletal muscle TnT gene in toad heart and the inactivation of cardiac TnT gene remains to be further investigated.

### Regulation of cardiac TnT expression via alternative RNA splicing

Multiple alternative splice forms are expressed from each of the three TnT isoform genes to add structural and functional variations that fine-tune muscle contractility. Alternative splice forms of cardiac TnT are expressed in a regulated pattern during embryonic and postnatal heart development, and are found in diseased hearts (Jin and Lin, 1988, 1989; Townsend et al., 1995; Ricchiuti and Apple, 1999). In addition to physiological or pathophysiological adaptations, aberrant splicing has been found to cause dilated cardiomyopathy in turkeys and dogs (Biesiadecki and Jin, 2002; Biesiadecki et al., 2002).

Mammalian cardiac TnT gene contains 17 exons including 3 alternatively spliced exons (exon 4, exon 5, and exon 13) (Jin et al., 1992, 1996) (Figure 3). Exon 4 and exon 5 encode segments in the N-terminal variable region, and exon 13 encodes a variable segment between the T1 and T2 functional domains (Jin et al., 1996). Multiple splice forms of cardiac TnT are expressed in mammalian hearts (Jin and Lin, 1988, 1989; Anderson et al., 1991; Jin et al., 1992, 1996). The alternative splicing pattern of cardiac TnT is synchronized in developing cardiac and skeletal muscle independent of functional demands (Jin, 1996). It was further demonstrated that the abundant cardiomyocytes present in the walls of developing and adult rat and mouse thoracic veins exhibit patterns of cardiac TnT alternative splice forms identical to that in the heart (Kracklauer et al., 2013; Liu et al., 2014). These findings strongly support that the regulation of cardiac TnT alternative splicing during development and differentiation is under systemic control rather than directly responding to functional demands or adaptation.

Combinations of alternative splicing of exons 4 and 5 in the N-terminal variable region yield four cardiac TnT isoforms differing in size and charge: TnT1 (all exon present), TnT2 (splice out exon 4), TnT3 (splice out exon 5), and TnT4 (splice out exon 4 and 5), numbered in the order of decreasing molecular weight (Gomes et al., 2002). *In vitro* studies showed that both TnT1 and TnT2 reduced the ability of troponin to inhibit force development and ATPase activity, causing less relaxation of fibers (Gomes et al., 2002). Whether the expression of cardiac TnT splice forms is altered and plays a role in failing heart is controversial and an area of active investigation (Anderson et al., 1991, 1992; Mesnard et al., 1995).

Abnormal splice isoforms of cardiac TnT have been reported. Turkeys with inherited dilated cardiomyopathy and heart failure have an aberrant splice-out of the normally conserved exon 8-encoded segment in cardiac TnT (Biesiadecki and Jin, 2002). Similar abnormality (splice out of the equivalent exon 7) has been found in cardiac TnT of dog, pig and cat, which also have high incidence of dilated cardiomyopathies (Biesiadecki et al., 2002). In the heart of adult guinea pig, exon 6 that is significantly larger than exon 7 is spliced out (Biesiadecki et al., 2002). Overexpression of exon 7-deleted cardiac TnT in the heart of transgenic mice impaired systolic function (Wei et al., 2010).

In addition to the deletion of exon 7, embryonic exon 5 is abnormally included at significant levels in adult cardiac TnT in dilated cardiomyopathy dogs (Biesiadecki et al., 2002). Although the continuing expression of this embryonic specific exon in the N-terminal region of cardiac TnT in adult heart may have a value to compensate for the abnormality of exon 7 deletion, we have shown that the heterogeneity of TnT in adult ventricular muscle due to the co-presence of more TnT variants reduces cardiac efficiency by desynchronizing the Ca^2+^-activation of thin filaments (Feng and Jin, 2010) (discussed in more details below).

Aberrant splicing of cardiac TnT also occurs in chronic stress conditions. Splice out of the exon 4-encoded segment was increased in failing human heart (Anderson et al., 1991, 1995), diabetic rat heart (Akella et al., 1995), and familial hypertrophic cardiomyopathy human hearts (Thierfelder et al., 1994). In a rabbit model of mild cardiac hypertrophy, cardiac TnT splicing shifted toward the fetal pattern (Chen et al., 1997). Further investigations are needed to understand the function as well as regulatory mechanisms of such potentially adaptive alternative splicing of cardiac TnT under stress conditions.

The mechanism for the aberrantly spliced cardiac TnT to produce dilated cardiomyopathy has been investigated. Different alternative splice forms of cardiac TnT are of different functional properties (Gomes et al., 2002). As mentioned above, a hypothesis is that chronic co-existence of TnT variants in adult heart would produce split Ca^2+^ sensitivity among troponins in the thin filament, which will desynchronize activation of ventricular muscle and decrease the efficiency of cardiac pumping (Feng and Jin, 2010). Different from skeletal muscles that normally express multiple TnT isoforms and splice forms corresponding to the function of tetanic contractions, the ventricular muscle is electrically activated as a syncytium to produce uniform rhythmic contractions. Consistently, only adult isoform of cardiac TnT is present in adult heart after the developmental switch (Jin and Lin, 1988; Jin, 1996), corresponding to a uniform sensitivity to Ca^2+^ activation.

Studies on transgenic mice demonstrated that co-existence of a non-mutant fast TnT and the endogenous cardiac TnT in adult heart significantly impaired contractile functions (Huang et al., 2008; Yu et al., 2012). Therefore, the desynchronized troponin activity, other than a mutant structure in TnT, imposed negative impacts on myocardial function. Further studies on transgenic mice expressing one or more functionally distinct cardiac TnT variants in addition to the endogenous normal adult cardiac TnT produced lower left ventricular pressure development, slower contractile and relaxation velocities, and decreased stroke volume as compared with wild-type controls, further supporting the hypothesis that coexistence of functionally different cardiac TnT variants in adult ventricular muscle reduces cardiac efficiency due to desynchronized thin filament activation (Feng and Jin, 2010).

The alternative splice forms found in avian and mammalian cardiac TnT are summarized in Table 1. The molecular mechanism that regulates alternative splicing of TnT remains to be fully understood, in which both *cis*-regulatory elements (Biesiadecki and Jin, 2002) and *trans*-regulatory factors (Ward and Cooper, 2010) have been suggested for roles in regulating the alternative splicing of cardiac TnT.

**Table 1:** **Physiological and abnormal alternative splice forms of cardiac TnT**.

**Splice forms**	**Physiological and pathophysiological significance**
Exon 4 splice-out	This exon encodes 4–5 amino acids and its alternative splicing results in relatively small change of N-terminal charge of cardiac TnT. Alternative splicing is normally found in rabbit, rat, mouse, and bovine hearts (Biesiadecki and Jin, 2002) and is increased in human heart failure (Anderson et al., 1991, 1995), human familial hypertrophic cardiomyopathy (Thierfelder et al., 1994), and the heart of diabetic rats (Akella et al., 1995).
Exon 5 splice-in	This exon encodes 9–10 mainly acidic amino acids. It is normally included in embryonic avian and mammalian cardiac TnT (Jin et al., 1992) and abnormally expressed in adult canine hearts of dilated cardiomyopathy (Biesiadecki et al., 2002). Its inclusion equips myofibrils with a higher tolerance to acidosis and higher Ca^2+^ sensitivity.
Exon 6 splice-out	This exon encodes 25 amino acids and its alternative splicing corresponds to a rather large structural change, abnormally occurring in adult Guinea pig hearts (Biesiadecki et al., 2002) with unknown functional effects.
Exon7 splice-out	This exon encodes 12 amino acids and is abnormally excluded in adult canine hearts with dilated cardiomyopathy, causing impairing systolic function (Biesiadecki et al., 2002).
Exon 8 splice-out	This exon encodes 12 amino acids equivalent to mammalian exon 7. Its abnormal exclusion in adult turkey hearts with dilated cardiomyopathy alters molecular conformation and binding affinity of cardiac TnT for cardiac TnI and tropomyosin (Biesiadecki and Jin, 2002).
Exon 13 splice-in/out	This exon encodes 2–3 amino acids. Its alternative splicing is independent of development and the functional significance is unknown (Jin et al., 1992).

### Phosphorylation modifications

Ser_2_ is a highly conserved residue in all three isoforms of avian and mammalian TnT (Jin et al., 2008) and is constitutively phosphorylated in cardiac TnT *in vivo* (Perry, 1998; Sancho Solis et al., 2008). Little is known regarding the kinase, regulation and functional significance of cardiac TnT Ser_2_ phosphorylation.

*In vitro* studies demonstrated that cardiac TnT could be phosphorylated by PKC at Thr_197_, Ser_201_, Thr_206_, and Thr_287_ (residue numbers in mouse cardiac TnT), which are located in the C-terminal region containing binding sites for TnI, TnC, and tropomyosin (Jideama et al., 1996). PKC-mediated phosphorylation of cardiac TnT has been shown to depress myofilament function, myocyte contractility, and ventricular pumping (Belin et al., 2007). PKC phosphorylation of cardiac TnT inhibited the Ca^2+^-stimulated Mg^2+^-ATPase activity without alteration of Ca^2+^-sensitivity (Noland and Kuo, 1993). When cardiac TnT was partially replaced with fast skeletal muscle TnT in transgenic mouse heart, which is not phosphorylated by PKC, the PKC-mediated depression of cardiac function was blunted (Montgomery et al., 2001). Studies with mutations at PKC phosphorylation sites supported the hypothesis that Thr_206_ is a regulatory site whose phosphorylation by PKCα or substitution with Glu to mimic phosphorylation significantly suppressed tension development, actomyosin Mg^2+^-ATPase activity, and myofilament Ca^2+^ sensitivity and cooperativity while Thr_197_, Ser_201_, and Thr_287_ had no significant effect (Sumandea et al., 2003).

Thr_206_ can also be phosphorylated by Raf-1, which links growth factor-dependent signaling to dynamic changes in cardiac contractile function (Pfleiderer et al., 2009). Ser_278_ and Thr_287_ of cardiac TnT were also found to be phosphorylated by Rho-dependent kinase (ROCK2), which inhibited tension development and ATPase activity in skinned fibers (Vahebi et al., 2005). Phosphorylation of cardiac TnT by ASK1 (a stress-activated kinase that has been implicated in TNFα and oxidant stress responses) at Thr_194_ and Ser_198_ has also been found with a decrease in cardiomyocyte contractility (He et al., 2003).

Protein phosphatase 1 (PP1) has been found to dephosphorylate cardiac TnT (Jideama et al., 2006). A coimmunoprecipitation study indicated that Pak1 (p21 activated kinase 1) is associated with cardiac TnT and regulates the phosphorylation level of cardiac TnT (Monasky et al., 2012). Hearts of Pak1 knockout mice showed a significant increase in TnT phosphorylation as compared with wild type controls (Ke et al., 2012). This modification may contribute to cardioprotection through Pak1 signaling and merits further investigation.

The phosphorylation of cardiac troponin could also be modulated by structure alterations. Deletion of Lys_210_ in cardiac TnT (ΔK210) decreased the phosphorylations of cardiac TnT by 30% and cardiac TnI by 46%, mainly at Ser_23/24_, *in vivo* as compared with wild-type controls (Sfichi-Duke et al., 2010). *In vitro* kinase assay indicated that ΔK210 increased phosphorylation propensity of Thr_203_ in cardiac TnT by three-fold, without changing Ser_23/24_ phosphorylation in cardiac TnI. Yeast two-hybrid studies indicated that cardiac TnT-ΔK210 bound stronger to cardiac TnI than that of wild type cardiac TnT (Sfichi-Duke et al., 2010), suggesting a possible explanation for cardiac TnT-ΔK210 mutation to correlate with dilated cardiomyopathy (Kamisago et al., 2000).

### Proteolytic regulations

#### Rapid degradation of non-myofilalemt associated TnT

Cardiac TnT has a half-life of 3.5 days *in vivo* (Martin, 1981) and non-myofilament-associated TnT is rapidly degraded in cardiomyocytes (Wang et al., 2005; Jeong et al., 2009). The potent proteolysis capacity in cardiomyocytes may be critical to maintaining the integrity of myofilament contractile apparatus as well as to protecting cardiomyocytes from the cytotoxicity of TnT fragment (Jeong et al., 2009). In the absence of myofilaments, the C-terminal and middle fragments of TnT effectively induced cell apoptosis (Jeong et al., 2009). A hypothesis is that a peak release of cardiac TnT or cardiac TnT fragments from myofilaments exceeding the protective capacity of the proteolytic degradation would result in cytotoxicity and cause the death of cardiomyocytes in myocardial ischemia-reperfusion injury. No apoptosis-effect of N-terminal variable region was observed (Jeong et al., 2009). Along this line, an *in vitro* study showed that cardiac TnT was cleaved by activated caspase 3 to remove the N-terminal 92 amino acids and resulted in contractile dysfunction before cell death (Communal et al., 2002).

#### Restrictive N-terminal truncation

Restrictive deletion of the N-terminal 71 residues of mouse cardiac TnT was found in hearts after ischemia reperfusion (Zhang et al., 2006) and left ventricular pressure overload *in vitro* (Feng et al., 2008a). Amino acid sequencing and protein fragment reconstruction determined that this restrictive N-terminal proteolysis selectively removes the entire N-terminal variable region but preserves the conserved core structure of cardiac TnT intact (Zhang et al., 2006). Myofilament associated μ-calpain is found to contribute the restrictive N-terminal truncation of cardiac TnT (Zhang et al., 2006).

The selective removal of the N-terminal variable region had no significant effect on the binding affinities of cardiac TnT for TnI and tropomyosin. This observation demonstrates that the N-terminal variable region is not essential for the core function of TnT, and the restrictive N-terminal truncation of cardiac TnT may be a regulatory mechanism. In contrast, extended deletion to remove the N-terminal 91 residues of mouse cardiac TnT including a segment of the conserved middle region weakened the binding to tropomyosin (Biesiadecki et al., 2007a) as well as increased the Ca^2+^ sensitivity of troponin (Sumandea et al., 2009).

The restrictive cleavage of cardiac TnT can be induced with calcium overloading. The level of N-terminal truncated cardiac TnT (cTnT-ND) increased in primary cultures of adult mouse cardiomyocytes upon ouabain-produced Ca^2+^ overload (Zhang et al., 2011b). No degradation of cardiac TnI, a known substrate of μ-calpain, was detected and no significant alteration of phosphorylation was seen in cardiac TnT when Ca^2+^ overload produced cTnT-ND (Zhang et al., 2011b). These observations support a hypothesis that the induction of cTnT-ND in calcium overload is neither due to elevated overall activity of μ-calpain nor phosphorylation level of cardiac TnT. On the other hand, the structure of N-terminal region *per se* exhibited a role in determining the restrictive μ-calpain proteolysis. Deletion of exon 7-encoded segment made cardiac TnT more sensitive to μ-calpain modification (Zhang et al., 2011b).

Although the restrictive removal of the N-terminal variable region of cardiac TnT does not abolish the core function of troponin (Hinkle et al., 1999; Biesiadecki et al., 2007a), it results in conformational changes of cardiac TnT, modulates TnT's binding affinity for TnI, TnC, and tropomyosin, and alters Ca^2+^ activation of actomyosin ATPase (Wang and Jin, 1998; Jin and Root, 2000; Jin et al., 2000; Gomes et al., 2002). Using pyrene-labeled tropomyosin, studies demonstrated that N-terminal truncated cardiac TnT strengthened the interactions between cardiac TnT_77−289_ and tropomyosin and stabilized cardiac myofilaments in a sub-maximally activated state (Chandra et al., 1999).

Consistent with the notion that the N-terminal variable region of TnT is non-essential but a regulatory structure, overexpression of cTnT-ND in transgenic mouse hearts effectively replaced endogenous intact cardiac TnT and supported cardiac function. The hearts showed a slightly but statistically significant decrease in contractile velocity, which resulted in elongated time of left ventricular rapid ejection phase especially at high afterload (Feng et al., 2008a). This effect produced a significant increase in stroke volume and demonstrated that the restrictive N-terminal truncation of cardiac TnT is a mechanism to modulate thin filament function and alter myosin cross-bridge kinetics, suggesting a novel approach to compensating for cardiac output in energetic crisis (Feng et al., 2008a).

The structural-functional domains of cardiac TnT, alternative spliced exons, phosphorylation sites, and proteolytic modifications are summarized in Figure 3.

## Learning from myopathic mutations in cardiac troponin

Numerous mutations in the genes encoding the three subunits of cardiac troponin have been found to cause cardiomyopathies. By increasing or decreasing Ca^2+^ sensitivity and force generation, troponin mutations contribute to the pathogeneses of inherited hypertrophic, restrictive and diastolic cardiomyopathies (Seidman and Seidman, 2001).

### Mutations in cardiac TnC

Mutations in cardiac/slow TnC account for approximately 0.4% of hypertrophic cardiomyopathy (Landstrom et al., 2008). L29Q mutation in *TNNC1* was the first such mutation identified (Hoffmann et al., 2001). L29Q mutation in cardiac TnC hindered the PKA-dependent phosphorylation of cardiac TnI at Ser_22_/Ser_23_, and reduced Ca^2+^ sensitivity of myofilaments in ATPase assays using reconstituted skeletal muscle myofibrils containing cardiac troponin (Schmidtmann et al., 2005). However, the same mutation increased Ca^2+^ sensitivity of force development when it was used to replace endogenous TnC in skinned mouse cardiomyocytes (Liang et al., 2008). This difference may have resulted from the different experimental conditions or the intrinsic difference between cardiac and skeletal muscles.

More missense mutations, for example A8V, C84Y, E134D, and D145E, in *TNNC1* have been reported in hypertrophic cardiomyopathies (Landstrom et al., 2008). Functional studies showed that A8V, C84Y, and D145E increased Ca^2+^ sensitivity of force development (Pinto et al., 2009). In addition, E59D, D75Y and G159D mutation in *TNNC1* are found in dilated cardiomyopathy patients. E59D and D75Y localized in the regulatory Ca^2+^ binding site II decrease myofilament calcium responsiveness (Lim et al., 2008). G159D is localized in a metal ion-binding site and, therefore, alters the function of troponin complex in regulating cardiac muscle contractility (Mogensen et al., 2004).

Besides altering Ca^2+^-induced conformational changes, mutations in cardiac TnC may alter molecular conformations involved in Ca^2+^ affinity and binding to cardiac TnI. An example is that L48Q substitution in human cardiac TnC made the hydrophobic core more exposed to cardiac TnI, thus increased the binding affinity for TnI (Wang et al., 2012a). Mutation A31S in *TNNC1* increases Ca^2+^ sensitivity, which may contribute to causing hypertrophic cardiomyopathy and arrhythmogenesis (Parvatiyar et al., 2012). Although G159D mutation in the C-lobe of cardiac TnC did not alter myofilament function, it blunted the myofilament desensitization induced by phosphorylation of cardiac TnI at Ser_23_/Ser_24_ (Finley et al., 1999; Biesiadecki et al., 2007b).

It is worth noting that mutations in the region of the inactive Ca^2+^-binding Site I of cardiac TnC are found at a much higher rate than that in the active Site II region (Hoffmann et al., 2001; Landstrom et al., 2008; Parvatiyar et al., 2012; Wang et al., 2012a). This observation suggests that most of the myopathic mutations in TnC fixed in the population are those causing relatively mild functional changes other than drastically destructive at critical sites of function, such as the sole regulatory site II of cardiac/slow TnC.

### Mutations in cardiac TnI

Cardiac TnI mutations account for approximately 5% of familial hypertrophic cardiomyopathy cases and at least 20 mutations of cardiac TnI have been reported to link to inherited restrictive cardiomyopathy with increased Ca^2+^ sensitivity and reduced ATPase activity and force development (Gomes and Potter, 2004; Gomes et al., 2005; Yumoto et al., 2005). These cardiac TnI mutations are mainly found in the inhibitory region and the C-terminal end segment, indicating functional relevance.

Cardiac TnI mutation R21C in the N-terminal extension associated with hypertrophic cardiomyopathy abolishes *in vivo* phosphorylation of Ser_23_/Ser_24_ (Wang et al., 2012a). The phenotype of this mutation supports the regulatory role of the N-terminal extension of cardiac TnI in diastolic function of the heart. Cardiac TnI mutation R145G found in familial hypertrophic cardiomyopathy is within the inhibitory region and alters the interaction of cardiac TnI with cardiac TnC. This mutation reduces the inhibition of actomyosin ATPase and thus increases Ca^2+^ sensitivity (Kimura et al., 1997; Lindhout et al., 2002). Lys_184_ deletion in the C-terminal region of cardiac TnI impairs relaxation kinetics and results in hypercontractility when overexpressed in mouse cardiomyocytes (Iorga et al., 2008). Similarly, transgenic mice over-expressing cardiac TnI with R193H mutation demonstrated impaired relaxation similar to that in human restrictive cardiomyopathy patient (Du et al., 2008). The negative impact of this cardiac TnI mutation on heart function showed a dose dependence (Li et al., 2013). These findings indicate the critical role of the C-terminal domain of TnI in muscle relaxation and diastolic function of the heart (Davis et al., 2007).

It is intriguing that over 94% of known disease-causing single nucleotide polymorphisms (SNPs) in cardiac TnI are located in the C-terminal half of the polypeptide chain (residues 128–210) (Palpant et al., 2010). This observation may indicate more stringent structure-function relationships in this region. Alternatively, this pattern may reflect that this region of TnI has a tolerance to structural modifications, allowing more mutations fixed in the population without reproductive lethality. These hypotheses require further investigation.

### Mutations in cardiac TnT

Mutations in cardiac TnT account for approximately 15% of familial hypertrophic cardiomyopathy cases. These mutations are characterized by severe myocardial disarray, relatively mild and often subclinical hypertrophy, and a high incidence of sudden cardiac death (Thierfelder et al., 1994; Watkins et al., 1995; Maron et al., 1996; Tardiff et al., 1998; Varnava et al., 2001; Sehnert et al., 2002). Together with the aberrant splicing of cardiac TnT found in turkey and dog cardiomyopathies, at least 52 point mutations of cardiac TnT have been reported to cause human heart diseases, including 50 missense mutations, one deletion and one splicing donor site mutation (Willott et al., 2010).

Mutations in different regions of cardiac TnT may contribute to the pathogenesis of cardiomyopathies via different mechanisms, including increasing the Ca^2+^-sensitivity of troponin complex, changing the binding affinity of cardiac TnT for cardiac TnI and the affinity of cardiac TnI for cardiac TnC, and perturbing the proper response of myocardial contraction to changes in pH (Harada and Potter, 2004).

Dilated cardiomyopathy is a major cause of heart failure, and genetic defects are a significant contributor to the disease (Lakdawala et al., 2012). Up to date, at least five cardiac TnT mutants: R131W (Mogensen et al., 2004), R141W (Li et al., 2001), R205L (Mogensen et al., 2004), Lys_210_ deletion (Kamisago et al., 2000), and D270N (Mogensen et al., 2004), all in the conserved core structure of cardiac TnT, are found to reduce Ca^2+^ sensitivity and produce phenotypes of dilated cardiomyopathy (Mirza et al., 2005).

It is worth noting that some troponin mutations have been reported with clinical phenotypes of more than one types of cardiomyopathy. A possible explanation is that while hypertrophic or restrictive cardiomyopathies may be the primary disease, dilated cardiomyopathy can develop in the later stages as the pathology progresses into congestive heart failure. It is also interesting to note that no clinical case of human cardiomyopathy mutation has been found in the N-terminal domain of cardiac TnT corresponding to the hypervariable region of TnT, which is naturally tolerant to structural variations (Jin et al., 2008; Wei and Jin, 2011).

As the mutations of different troponin subunits have different functional impacts, their combined phenotypes may indicate structural and functional relationships among the troponin subunits. For example, a mutation R111C is found in cardiac TnI of wild turkeys co-existing with the dilated cardiomyopathy-related aberrantly splice-out of exon 8 in cardiac TnT (Biesiadecki et al., 2004). By lowering the binding affinity of cardiac TnI for the mutant cardiac TnT that showed increased affinity for TnI, mutually compensatory effects were observed. While the mouse counterparts, cardiac TnI-K118C mutation and exon 7-deleted cardiac TnT, each alone has dominant negative phenotypes in transgenic mice, double transgenic mouse hearts co-expressing cardiac TnI-K118C mutation and exon 7-deleted cardiac TnT showed that the systolic abnormality of cardiac TnT exon 7 deletion and the diastolic abnormality of cardiac TnI-K118C mutation mutually canceled each other (Wei et al., 2010).

Another example is that the S69D and D73N mutations of cardiac TnC corrected the abnormal Ca^2+^ sensitivity increased by cardiac TnI-R192H mutation or ischemia-induced C-terminal truncation (cTnI_1−192_) of cardiac TnI (Liu et al., 2012).

**Figure 4:**
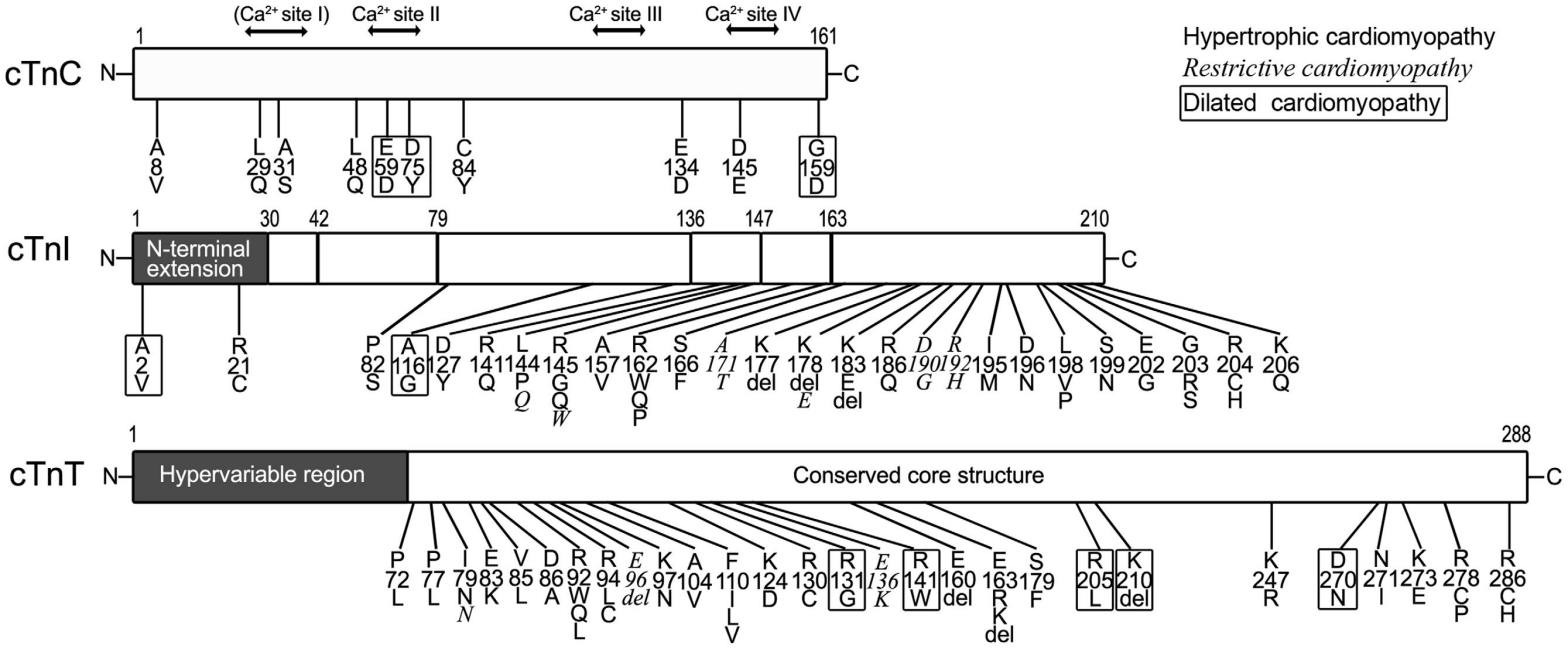
**Myopathic mutations in troponin subunits**. Amino acid substitutions and deletions found in human cardiomyopathies are indicated on the linear maps of cardiac TnC (cTnC, NP_003271.1), cardiac TnI (cTnI, NP_000354.4), and cardiac TnT (cTnT, NP_001263276.1). Different fonts were used to indicate that each of the mutations causes hypertrophic, restrictive (Italic), and dilated (boxed) cardiomyopathies. It is worth noting that no myopathic mutation was found in the N-terminal hypervariable region of cardiac TnT. The residue #s are counted including Met1.

Figure 4 summarizes most of the characterized human cardiomyopathic mutations found in cardiac TnC, cardiac TnI and cardiac TnT.

## Summary and perspective remarks

The TnC, TnI, and TnT subunits of cardiac troponin function interactively as regulators of myofilament activation and force generation. Based on biochemical, biophysical, physiological and pathophysiological studies, mounting evidence for the molecular evolution, gene regulation, alternative splicing, and posttranslational modifications of cardiac troponin subunits has laid a solid foundation for understanding their structural diversity, structure-function relationships, adaptive regulations, and pathogenic mutations. For troponin's central role in muscle thin filament regulation and contractility, further elucidation of troponin structure and function will powerfully forward the prevention, diagnosis, and treatment of heart diseases.

In order to advance troponin research and translate the knowledge into clinical applications, there are several important questions remain to be answered. With modern molecular engineering methodology, it is important and feasible to fine map the interaction sites between troponin subunits and the allosteric and conformational relationships that are essential in regulation of cardiac muscle contraction. Alternative splicing is an important regulatory pathway of cardiac TnT and aberrant splicing of it has close relation with cardiomyopathy. However, the mechanism of cardiac TnT expression via alternative splicing is not well understood. The cell signaling pathway that controls RNA splicing and the production of cardiac TnT variants remains to be investigated. Restrictive N-terminal truncations of cardiac TnI and cardiac TnT are novel posttranslational regulatory mechanisms that have potent roles in cardiac adaptation to stress conditions. However, the cellular mechanisms that induce restrictive N-terminal truncations of cardiac TnI and cardiac TnT have not been established. It is worth noting that no single stress condition has been found to be able to produce restrictive N-terminal truncations of both cardiac TnI and cardiac TnT, indicating distinct mechanisms in the posttranslational regulation of the two troponin subunits that are structurally and functionally closely related. It is also an intriguing observation that the N-terminal truncation of cardiac TnI selectively enhances diastolic function whereas the N-terminal truncation of cardiac TnT selectively reduces systolic velocity of the hearts. To understand how structural modifications of the two subunits of troponin regulate muscle contraction and relaxation in a highly selective manner would lead to development of new therapeutic approaches for the treatments systolic and diastolic heart failures.

Continued in depth research is required to answer these and new emerging questions toward the goal of fully understanding the function of troponin in cardiac muscle contraction in order to improve the treatment and prevention of myocardial diseases and heart failure.

### Conflict of interest statement

The authors declare that the research was conducted in the absence of any commercial or financial relationships that could be construed as a potential conflict of interest.

## References

[B1] Akella A. B. Ding X. L. Cheng R. Gulati J. (1995). Diminished Ca^2+^ sensitivity of skinned cardiac muscle contractility coincident with troponin T-band shifts in the diabetic rat. Circ. Res. 76, 600–606 10.1161/01.RES.76.4.600 7534660

[B2] Akhter S. Bueltmann K. Jr. Huang X. Jin J. P. (2014). Restrictive cardiomyopathy mutations demonstrate functions of the C-terminal end-segment of troponin I. Arch. Biochem. Biophys. [Epub ahead of print]. 10.1016/j.abb.2013.12.001 24326031

[B3] Akhter S. Zhang Z. Jin J. P. (2012). The heart-specific NH2-terminal extension regulates the molecular conformation and function of cardiac troponin I. Am. J. Physiol. Heart Circ. Physiol. 302, H923–H933 10.1152/ajpheart.00637.2011 22140044 PMC3322736

[B4] Anderson P. A. Greig A. Mark T. M. Malouf N. N. Oakeley A. E. Ungerleider R. M. (1995). Molecular basis of human cardiac troponin T isoforms expressed in the developing, adult, and failing heart. Circ. Res. 76, 681–686 10.1161/01.RES.76.4.681 7534662

[B5] Anderson P. A. Malouf N. N. Oakeley A. E. Pagani E. D. Allen P. D. (1991). Troponin T isoform expression in humans. A comparison among normal and failing adult heart, fetal heart, and adult and fetal skeletal muscle. Circ. Res. 69, 1226–1233 10.1161/01.RES.69.5.1226 1934353

[B6] Anderson P. A. Malouf N. N. Oakeley A. E. Pagani E. D. Allen P. D. (1992). Troponin T isoform expression in the normal and failing human left ventricle: a correlation with myofibrillar ATPase activity. Basic Res. Cardiol. 87(Suppl. 1), 117–127 1386729 10.1007/978-3-642-72474-9_10

[B7] Arteaga G. M. Palmiter K. A. Leiden J. M. Solaro R. J. (2000). Attenuation of length dependence of calcium activation in myofilaments of transgenic mouse hearts expressing slow skeletal troponin I. J. Physiol. 526(Pt 3), 541–549 10.1111/j.1469-7793.2000.t01-1-00541.x 10922006 PMC2270032

[B8] Barbato J. C. Huang Q. Q. Hossain M. M. Bond M. Jin J. P. (2005). Proteolytic N-terminal truncation of cardiac troponin I enhances ventricular diastolic function. J. Biol. Chem. 280, 6602–6609 10.1074/jbc.M408525200 15611140

[B9] Bardswell S. C. Cuello F. Rowland A. J. Sadayappan S. Robbins J. Gautel M. (2010). Distinct sarcomeric substrates are responsible for protein kinase D-mediated regulation of cardiac myofilament Ca^2+^ sensitivity and cross-bridge cycling. J. Biol. Chem. 285, 5674–5682 10.1074/jbc.M109.066456 20018870 PMC2820795

[B10] Belin R. J. Sumandea M. P. Allen E. J. Schoenfelt K. Wang H. Solaro R. J. (2007). Augmented protein kinase C-α–induced myofilament protein phosphorylation contributes to myofilament dysfunction in experimental congestive heart failure. Circ. Res. 101, 195–204 10.1161/CIRCRESAHA.107.148288 17556659

[B11] Biesiadecki B. J. Chong S. M. Nosek T. M. Jin J. P. (2007a). Troponin T core structure and the regulatory NH2-terminal variable region. Biochemistry 46, 1368–1379 10.1021/bi061949m 17260966 PMC1794682

[B12] Biesiadecki B. J. Elder B. D. Yu Z. B. Jin J. P. (2002). Cardiac troponin T variants produced by aberrant splicing of multiple exons in animals with high instances of dilated cardiomyopathy. J. Biol. Chem. 277, 50275–50285 10.1074/jbc.M206369200 12377784

[B13] Biesiadecki B. J. Jin J. P. (2002). Exon skipping in cardiac troponin T of turkeys with inherited dilated cardiomyopathy. J. Biol. Chem. 277, 18459–18468 10.1074/jbc.M200788200 11886865

[B14] Biesiadecki B. J. Kobayashi T. Walker J. S. John Solaro R. De Tombe P. P. (2007b). The troponin C G159D mutation blunts myofilament desensitization induced by troponin I Ser23/24 phosphorylation. Circ. Res. 100, 1486–1493 10.1161/01.RES.0000267744.92677.7f 17446435

[B15] Biesiadecki B. J. Schneider K. L. Yu Z. B. Chong S. M. Jin J. P. (2004). An R111C polymorphism in wild turkey cardiac troponin I accompanying the dilated cardiomyopathy-related abnormal splicing variant of cardiac troponin T with potentially compensatory effects. J. Biol. Chem. 279, 13825–13832 10.1074/jbc.M314225200 14736877

[B16] Biesiadecki B. J. Tachampa K. Yuan C. Jin J. P. De Tombe P. P. Solaro R. J. (2010). Removal of the cardiac troponin I N-terminal extension improves cardiac function in aged mice. J. Biol. Chem. 285, 19688–19698 10.1074/jbc.M109.086892 20410305 PMC2885247

[B17] Blumenschein T. M. A. Tripet B. P. Hodges R. S. Sykes B. D. (2001). Mapping the interacting regions between troponins T and C: binding of TnT and TnI peptides to TnC and NMR mapping of the TnT-binding site on TnC. J. Biol. Chem. 276, 36606–36612 10.1074/jbc.M105130200 11473120

[B18] Bodor G. S. Oakeley A. E. Allen P. D. Crimmins D. L. Ladenson J. H. Anderson P. A. W. (1997). Troponin I phosphorylation in the normal and failing adult human heart. Circulation 96, 1495–1500 10.1161/01.CIR.96.5.1495 9315537

[B19] Boral M. C. Deb C. (1970). Seasonal changes in body fluids and haematology in toad Bufo melanostictus a poikilothermic cold torpor. Proc. Ind. Natl. Sci. Acad. 36, 369–379

[B20] Burkart E. M. Arteaga G. M. Sumandea M. P. Prabhakar R. Wieczorek D. F. Solaro R. J. (2003a). Altered signaling surrounding the C-lobe of cardiac troponin C in myofilaments containing an alpha-tropomyosin mutation linked to familial hypertrophic cardiomyopathy. J. Mol. Cell. Cardiol. 35, 1285 10.1016/S0022-2828(03)00240-2 14519438

[B21] Burkart E. M. Sumandea M. P. Kobayashi T. Nili M. Martin A. F. Homsher E. (2003b). Phosphorylation or glutamic acid substitution at protein kinase C sites on cardiac troponin I differentially depress myofilament tension and shortening velocity. J. Biol. Chem. 278, 11265–11272 10.1074/jbc.M210712200 12551921

[B22] Buscemi N. Foster D. B. Neverova I. Van Eyk J. E. (2002). p21-activated kinase increases the calcium sensitivity of rat triton-skinned cardiac muscle fiber bundles via a mechanism potentially involving novel phosphorylation of troponin I. Circ. Res. 91, 509–516 10.1161/01.RES.0000035246.27856.53 12242269

[B23] Chandra M. Montgomery D. E. Kim J. J. Solaro R. J. (1999). The N-terminal region of troponin T is essential for the maximal activation of rat cardiac myofilaments. J. Mol. Cell. Cardiol. 31, 867–880 10.1006/jmcc.1999.0928 10329214

[B24] Chen Z. Higashiyama A. Yaku H. Bell S. Fabian J. Watkins M. W. (1997). Altered expression of troponin T isoforms in mild left ventricular hypertrophy in the rabbit. J. Mol. Cell. Cardiol. 29, 2345 10.1006/jmcc.1997.0468 9299358

[B25] Chong S. M. Jin J. P. (2009). To investigate protein evolution by detecting suppressed epitope structures. J. Mol. Evol. 68, 448–460 10.1007/s00239-009-9202-0 19365646 PMC2752406

[B26] Collins J. H. (1991). Myosin light chains and troponin C: structural and evolutionary relationships revealed by amino acid sequence comparisons. J. Muscle Res. Cell Motil. 12, 3–25 10.1007/BF01781170 2050809

[B27] Communal C. Sumandea M. De Tombe P. Narula J. Solaro R. J. Hajjar R. J. (2002). Functional consequences of caspase activation in cardiac myocytes. Proc. Natl. Acad. Sci. U.S.A. 99, 6252–6256 10.1073/pnas.092022999 11972044 PMC122935

[B28] Cuello F. Bardswell S. C. Haworth R. S. Yin X. Lutz S. Wieland T. (2007). Protein kinase D selectively targets cardiac troponin I and regulates myofilament Ca^2+^ sensitivity in ventricular myocytes. Circ. Res. 100, 864–873 10.1161/01.RES.0000260809.15393.fa 17322173

[B29] Davis J. Wen H. Edwards T. Metzger J. M. (2007). Thin filament disinhibition by restrictive cardiomyopathy mutant R193H troponin I induces Ca^2+^-independent mechanical tone and acute myocyte remodeling. Circ. Res. 100, 1494–1502 10.1161/01.RES.0000268412.34364.50 17463320

[B30] Deb C. Chatterjee S. Boral M. C. (1974). Body fluid and hematological changes in toads following heat exposure. Am. J. Physiol. 226, 408–410 4811198 10.1152/ajplegacy.1974.226.2.408

[B31] Di Lisa F. De Tullio R. Salamino F. Barbato R. Melloni E. Siliprandi N. (1995). Specific degradation of troponin T and I by mu-calpain and its modulation by substrate phosphorylation. Biochem. J. 308, 57 7755588 10.1042/bj3080057 PMC1136842

[B32] Du J. Liu J. Feng H. Z. Hossain M. M. Gobara N. Zhang C. (2008). Impaired relaxation is the main manifestation in transgenic mice expressing a restrictive cardiomyopathy mutation, R193H, in cardiac TnI. Am. J. Physiol. Heart Circ. Physiol. 294, H2604–H2613 10.1152/ajpheart.91506.2007 18408133 PMC2769498

[B33] Farah C. S. Miyamoto C. A. Ramos C. H. Da Silva A. C. Quaggio R. B. Fujimori K. (1994). Structural and regulatory functions of the NH2- and COOH-terminal regions of skeletal muscle troponin I. J. Biol. Chem. 269, 5230–5240 8106506

[B35] Feng H. Z. Biesiadecki B. J. Yu Z. B. Hossain M. M. Jin J. P. (2008a). Restricted N-terminal truncation of cardiac troponin T: a novel mechanism for functional adaptation to energetic crisis. J. Physiol. 586, 3537–3550 10.1113/jphysiol.2008.153577 18556368 PMC2538805

[B36] Feng H. Z. Chen M. Weinstein L. S. Jin J. P. (2008b). Removal of the N-terminal extension of cardiac troponin I as a functional compensation for impaired myocardial beta-adrenergic signaling. J. Biol. Chem. 283, 33384–33393 10.1074/jbc.M803302200 18815135 PMC2586242

[B37] Feng H. Z. Chen X. Hossain M. M. Jin J. P. (2012). Toad heart utilizes exclusively slow skeletal muscle troponin T: an evolutionary adaptation with potential functional benefits. J. Biol. Chem. 287, 29753–29764 10.1074/jbc.M112.373191 22778265 PMC3436204

[B38] Feng H. Z. Hossain M. M. Huang X. P. Jin J. P. (2009a). Myofilament incorporation determines the stoichiometry of troponin I in transgenic expression and the rescue of a null mutation. Arch. Biochem. Biophys. 487, 36–41 10.1016/j.abb.2009.05.001 19433057 PMC2752407

[B34] Feng H. Z. Jin J. P. (2010). Coexistence of cardiac troponin T variants reduces heart efficiency. Am. J. Physiol. Heart Circ. Physiol. 299, H97–H105 10.1152/ajpheart.01105.2009 20418479 PMC2904141

[B39] Feng H. Z. Wei B. Jin J. P. (2009b). Deletion of a genomic segment containing the cardiac troponin I gene knocks down expression of the slow troponin T gene and impairs fatigue tolerance of diaphragm muscle. J. Biol. Chem. 284, 31798–31806 10.1074/jbc.M109.020826 19797054 PMC2797250

[B40] Feng J. Schaus B. J. Fallavollita J. A. Lee T. C. Canty J. M. Jr. (2001). Preload induces troponin I degradation independently of myocardial ischemia. Circulation 103, 2035–2037 10.1161/01.CIR.103.16.2035 11319190

[B41] Fentzke R. C. Buck S. H. Patel J. R. Lin H. Wolska B. M. Stojanovic M. O. (1999). Impaired cardiomyocyte relaxation and diastolic function in transgenic mice expressing slow skeletal troponin I in the heart. J. Physiol. 517, 143–157 10.1111/j.1469-7793.1999.0143z.x 10226156 PMC2269324

[B42] Finley N. Abbott M. B. Abusamhadneh E. Gaponenko V. Dong W. Gasmi-Seabrook G. (1999). NMR analysis of cardiac troponin C-troponin I complexes: effects of phosphorylation. FEBS Lett. 453, 107–112 10.1016/S0014-5793(99)00693-6 10403385

[B43] Flicker P. F. Phillips G. N. Jr. Cohen C. (1982). Troponin and its interactions with tropomyosin: an electron microscope study. J. Mol. Biol. 162, 495–501 10.1016/0022-2836(82)90540-X 7161805

[B44] Foster D. B. Noguchi T. Vanburen P. Murphy A. M. Van Eyk J. E. (2003). C-terminal truncation of cardiac troponin I causes divergent effects on ATPase and force implications for the pathophysiology of myocardial stunning. Circ. Res. 93, 917–924 10.1161/01.RES.0000099889.35340.6F 14551240

[B45] Galiñska A. Hatch V. Craig R. Murphy A. M. Van Eyk J. E. Wang C. L. A. (2010). The C terminus of cardiac troponin I stabilizes the Ca^2+^-activated state of tropomyosin on actin filaments. Circ. Res. 106, 705–711 10.1161/CIRCRESAHA.109.210047 20035081 PMC2834238

[B46] Gomes A. V. Guzman G. Zhao J. Potter J. D. (2002). Cardiac troponin T isoforms affect the Ca^2+^ sensitivity and inhibition of force development. Insights into the role of troponin T isoforms in the heart. J. Biol. Chem. 277, 35341–35349 10.1074/jbc.M204118200 12093807

[B47] Gomes A. V. Liang J. Potter J. D. (2005). Mutations in human cardiac troponin I that are associated with restrictive cardiomyopathy affect basal ATPase activity and the calcium sensitivity of force development. J. Biol. Chem. 280, 30909–30915 10.1074/jbc.M500287200 15961398

[B48] Gomes A. V. Potter J. D. (2004). Cellular and molecular aspects of familial hypertrophic cardiomyopathy caused by mutations in the cardiac troponin I gene. Mol. Cell. Biochem. 263, 99–114 10.1023/B:MCBI.0000041852.42291.aa 15524171

[B49] Gordon A. Homsher E. Regnier M. (2000). Regulation of contraction in striated muscle. Physiol. Rev. 80, 853–924 10747208 10.1152/physrev.2000.80.2.853

[B50] Grabarek Z. Tao T. Gergely J. (1992). Molecular mechanism of troponin-C function. J. Muscle Res. Cell Motil. 13, 383–393 10.1007/BF01738034 1401036

[B51] Greaser M. Gergely J. (1971). Reconstitution of troponin activity from three protein components. J. Biol. Chem. 246, 4226–4233 4253596

[B52] Guo X. Wattanapermpool J. Palmiter K. A. Murphy A. M. Solaro R. J. (1994). Mutagenesis of cardiac troponin I. Role of the unique NH2-terminal peptide in myofilament activation. J. Biol. Chem. 269, 15210–15216 8195157

[B53] Harada K. Potter J. D. (2004). Familial hypertrophic cardiomyopathy mutations from different functional regions of Troponin T result in different effects on the pH and Ca^2+^ sensitivity of cardiac muscle contraction. J. Biol. Chem. 279, 14488–14495 10.1074/jbc.M309355200 14722098

[B54] Hastings K. (1997). Molecular evolution of the vertebrate troponin I gene family. Cell Struct. Funct. 22:205 10.1247/csf.22.205 9113408

[B55] Haworth R. S. Cuello F. Herron T. J. Franzen G. Kentish J. C. Gautel M. (2004). Protein kinase D is a novel mediator of cardiac troponin I phosphorylation and regulates myofilament function. Circ. Res. 95, 1091–1099 10.1161/01.RES.0000149299.34793.3c 15514163

[B56] He X. Liu Y. Sharma V. Dirksen R. T. Waugh R. Sheu S. S. (2003). ASK1 associates with troponin T and induces troponin T phosphorylation and contractile dysfunction in cardiomyocytes. Am. J. Pathol. 163, 243–251 10.1016/S0002-9440(10)63647-4 12819028 PMC1868161

[B57] Heeley D. Golosinska K. Smillie L. B. (1987). The effects of troponin T fragments T1 and T2 on the binding of nonpolymerizable tropomyosin to F-actin in the presence and absence of troponin I and troponin C. J. Biol. Chem. 262, 9971–9978 3611073

[B58] Herzberg O. James M. N. G. (1985). Structure of the calcium regulatory muscle protein troponin-C at 2.8 Å resolution. Nature 313, 653–659 10.1038/313653a0 3974698

[B59] Hinkle A. Goranson A. Butters C. A. Tobacman L. S. (1999). Roles for the troponin tail domain in thin filament assembly and regulation: a deletion study of cardiac troponin T. J. Biol. Chem. 274, 7157–7164 10.1074/jbc.274.11.7157 10066775

[B60] Hoffmann B. Schmidt-Traub H. Perrot A. Osterziel K. J. Gessner R. (2001). First mutation in cardiac troponin C, L29Q, in a patient with hypertrophic cardiomyopathy. Hum. Mutat. 17, 524–524 10.1002/humu.1143 11385718

[B61] Howarth J. W. Meller J. Solaro R. J. Trewhella J. Rosevear P. R. (2007). Phosphorylation-dependent conformational transition of the cardiac specific N-extension of troponin I in cardiac troponin. J. Mol. Biol. 373, 706–722 10.1016/j.jmb.2007.08.035 17854829

[B62] Huang Q. Q. Feng H. Z. Liu J. Du J. Stull L. B. Moravec C. S. (2008). Co-expression of skeletal and cardiac troponin T decreases mouse cardiac function. Am. J. Physiol. Cell Physiol. 294, C213–C222 10.1152/ajpcell.00146.2007 17959729

[B63] Iorga B. Blaudeck N. Solzin J. Neulen A. Stehle I. Davila A. J. L. (2008). Lys184 deletion in troponin I impairs relaxation kinetics and induces hypercontractility in murine cardiac myofibrils. Cardiovasc. Res. 77, 676–686 10.1093/cvr/cvm113 18096573

[B64] Jeong E. M. Wang X. Xu K. Hossain M. M. Jin J. P. (2009). Nonmyofilament-associated troponin T fragments induce apoptosis. Am. J. Physiol. Heart Circ. Physiol. 297, H283–H292 10.1152/ajpheart.01200.2008 19395545 PMC2711745

[B65] Jideama N. M. Crawford B. H. Hussain A. K. M. A. Raynor R. L. (2006). Dephosphorylation specificities of protein phosphatase for cardiac troponin I, troponin T, and sites within troponin T. Int. J. Biol. Sci. 2:1 10.7150/ijbs.2.1 16585947 PMC1415850

[B66] Jideama N. M. Noland T. A. Jr. Raynor R. L. Blobe G. C. Fabbro D. Kazanietz M. G. (1996). Phosphorylation specificities of protein kinase C isozymes for bovine cardiac troponin I and troponin T and sites within these proteins and regulation of myofilament properties. J. Biol. Chem. 271, 23277–23283 10.1074/jbc.271.38.23277 8798526

[B67] Jin J. P. (1996). Alternative RNA splicing-generated cardiac troponin T isoform switching: a non-heart-restricted genetic programming synchronized in developing cardiac and skeletal muscles. Biochem. Biophys. Res. Commun. 225, 883–889 10.1006/bbrc.1996.1267 8780706

[B68] Jin J. P. Chen A. Ogut O. Huang Q. Q. (2000). Conformational modulation of slow skeletal muscle troponin T by an NH(2)-terminal metal-binding extension. Am. J. Physiol. Cell Physiol. 279, C1067–C1077 11003587 10.1152/ajpcell.2000.279.4.C1067

[B69] Jin J. P. Chong S. M. (2010). Localization of the two tropomyosin-binding sites of troponin T. Arch. Biochem. Biophys. 500, 144–150 10.1016/j.abb.2010.06.001 20529660 PMC2904419

[B70] Jin J. P. Huang Q. Q. Yeh H. I. Lin J. J. (1992). Complete nucleotide sequence and structural organization of rat cardiac troponin T gene. A single gene generates embryonic and adult isoforms via developmentally regulated alternative splicing. J. Mol. Biol. 227, 1269–1276 10.1016/0022-2836(92)90540-Z 1433301

[B71] Jin J. P. Lin J. J. (1988). Rapid purification of mammalian cardiac troponin T and its isoform switching in rat hearts during development. J. Biol. Chem. 263, 7309–7315 3366782

[B72] Jin J. P. Lin J. J. (1989). Isolation and characterization of cDNA clones encoding embryonic and adult isoforms of rat cardiac troponin T. J. Biol. Chem. 264, 14471–14477 2760070

[B73] Jin J. P. Root D. D. (2000). Modulation of troponin T molecular conformation and flexibility by metal ion binding to the NH2-terminal variable region. Biochemistry 39, 11702–11713 10.1021/bi9927437 10995238

[B74] Jin J. P. Smillie L. B. (1994). An unusual metal-binding cluster found exclusively in the avian breast muscle troponin T of Galliformes and Craciformes. FEBS Lett. 341, 135–140 10.1016/0014-5793(94)80256-4 8137914

[B75] Jin J. P. Wang J. Zhang J. (1996). Expression of cDNAs encoding mouse cardiac troponin T isoforms: characterization of a large sample of independent clones. Gene 168, 217–221 10.1016/0378-1119(95)00803-9 8654947

[B76] Jin J. P. Yang F. W. Yu Z. B. Ruse C. I. Bond M. Chen A. (2001). The highly conserved COOH terminus of troponin I forms a Ca^2+^-modulated allosteric domain in the troponin complex. Biochemistry 40, 2623–2631 10.1021/bi002423j 11327886

[B77] Jin J. P. Zhang Z. Bautista J. A. (2008). Isoform diversity, regulation, and functional adaptation of troponin and calponin. Crit. Rev. Eukaryot Gene Expr. 18, 93–124 10.1615/CritRevEukarGeneExpr.v18.i2.10 18304026

[B78] Kamisago M. Sharma S. D. Depalma S. R. Solomon S. Sharma P. McDonough B. (2000). Mutations in sarcomere protein genes as a cause of dilated cardiomyopathy. N. Engl. J. Med. 343, 1688–1696 10.1056/NEJM200012073432304 11106718

[B79] Kawasaki H. Nakayama S. Kretsinger R. (1998). Classification and evolution of EF-hand proteins. Biometals 11, 277–295 10.1023/A:1009282307967 10191494

[B80] Ke Y. Lei M. Wang X. Solaro R. J. (2012). Novel roles of PAK1 in the heart. Cell. Logist. 2, 89–94 10.4161/cl.21497 23162741 PMC3490967

[B81] Ke Y. Wang L. Pyle W. G. De Tombe P. P. Solaro R. J. (2004). Intracellular localization and functional effects of P21-activated kinase-1 (Pak1) in cardiac myocytes. Circ. Res. 94, 194–200 10.1161/01.RES.0000111522.02730.56 14670848

[B82] Kim S. J. Kudej R. K. Yatani A. Kim Y. K. Takagi G. Honda R. (2001). A novel mechanism for myocardial stunning involving impaired Ca^2+^ handling. Circ. Res. 89, 831–837 10.1161/hh2101.098547 11679414

[B83] Kimura A. Harada H. Park J. E. Nishi H. Satoh M. Takahashi M. (1997). Mutations in the cardiac troponin I gene associated with hypertrophic cardiomyopathy. Nat. Genet. 16, 379–382 10.1038/ng0897-379 9241277

[B84] Kirkpatrick K. P. Robertson A. S. Klaiman J. M. Gillis T. E. (2011). The influence of trout cardiac troponin I and PKA phosphorylation on the Ca^2+^ affinity of the cardiac troponin complex. J. Exp. Biol. 214, 1981–1988 10.1242/jeb.052860 21613513

[B85] Kobayashi T. Yang X. Walker L. A. Van Breemen R. B. Solaro R. J. (2005). A non-equilibrium isoelectric focusing method to determine states of phosphorylation of cardiac troponin I: identification of Ser-23 and Ser-24 as significant sites of phosphorylation by protein kinase C. J. Mol. Cell. Cardiol. 38, 213–218 10.1016/j.yjmcc.2004.10.014 15623438

[B86] Kooij V. Stienen G. J. M. Van Der Velden J. (2011). The role of protein kinase C-mediated phosphorylation of sarcomeric proteins in the heart—detrimental or beneficial? Biophys. Rev. 3, 107–117 10.1007/s12551-011-0050-y PMC5425667 28510060

[B87] Kracklauer M. P. Feng H. Z. Jiang W. Lin J. L. Lin J. J. Jin J. P. (2013). Discontinuous thoracic venous cardiomyocytes and heart exhibit synchronized developmental switch of troponin isoforms. FEBS J. 280, 880–891 10.1111/febs.12076 23176202 PMC3734956

[B88] Lakdawala N. K. Thune J. J. Colan S. D. Cirino A. L. Farrohi F. Rivero J. (2012). Subtle abnormalities in contractile function are an early manifestation of sarcomere mutations in dilated cardiomyopathy. Circ. Cardiovasc. Genet. 5, 503–510 10.1161/CIRCGENETICS.112.962761 22949430 PMC3646896

[B89] Landstrom A. P. Parvatiyar M. S. Pinto J. R. Marquardt M. L. Bos J. M. Tester D. J. (2008). Molecular and functional characterization of novel hypertrophic cardiomyopathy susceptibility mutations in TNNC1-encoded troponin C. J. Mol. Cell. Cardiol. 45, 281–288 10.1016/j.yjmcc.2008.05.003 18572189 PMC2627482

[B90] Layland J. Li J. M. Shah A. M. (2002). Role of cyclic GMP-dependent protein kinase in the contractile response to exogenous nitric oxide in rat cardiac myocytes. J. Physiol. 540, 457–467 10.1113/jphysiol.2001.014126 11956336 PMC2290258

[B91] Layland J. Solaro R. J. Shah A. M. (2005). Regulation of cardiac contractile function by troponin I phosphorylation. Cardiovasc. Res. 66, 12–21 10.1016/j.cardiores.2004.12.022 15769444

[B92] Li D. Czernuszewicz G. Z. Gonzalez O. Tapscott T. Karibe A. Durand J.-B. (2001). Novel cardiac troponin T mutation as a cause of familial dilated cardiomyopathy. Circulation 104, 2188–2193 10.1161/hc4301.098285 11684629

[B93] Li M. X. Wang X. Lindhout D. A. Buscemi N. Van Eyk J. E. Sykes B. D. (2003). Phosphorylation and mutation of human cardiac troponin I deferentially destabilize the interaction of the functional regions of troponin I with troponin C. Biochemistry 42, 14460–14468 10.1021/bi035408y 14661957

[B94] Li M. X. Wang X. Sykes B. D. (2004). Structural based insights into the role of troponin in cardiac muscle pathophysiology. J. Muscle Res. Cell Motil. 25, 559–579 10.1007/s10974-004-5879-2 15711886

[B95] Li Y. Charles P. Y. Nan C. Pinto J. R. Wang Y. Liang J. (2010). Correcting diastolic dysfunction by Ca^2+^ desensitizing troponin in a transgenic mouse model of restrictive cardiomyopathy. J. Mol. Cell. Cardiol. 49, 402–411 10.1016/j.yjmcc.2010.04.017 20580639 PMC5394742

[B96] Li Y. Zhang L. Jean-Charles P. Y. Nan C. Chen G. Tian J. (2013). Dose-dependent diastolic dysfunction and early death in a mouse model with cardiac troponin mutations. J. Mol. Cell. Cardiol. 62, 227–236 10.1016/j.yjmcc.2013.06.007 23810866 PMC5394738

[B97] Liang B. Chung F. Qu Y. Pavlov D. Gillis T. E. Tikunova S. B. (2008). Familial hypertrophic cardiomyopathy-related cardiac troponin C mutation L29Q affects Ca^2+^ binding and myofilament contractility. Physiol. Genomics 33, 257–266 10.1152/physiolgenomics.00154.2007 18285522

[B98] Lim C. C. Yang H. Yang M. Wang C. K. Shi J. Berg E. A. (2008). A novel mutant cardiac troponin C disrupts molecular motions critical for calcium binding affinity and cardiomyocyte contractility. Biophys. J. 94, 3577–3589 10.1529/biophysj.107.112896 18212018 PMC2292379

[B99] Lindhout D. A. Li M. X. Schieve D. Sykes B. D. (2002). Effects of T142 phosphorylation and mutation R145G on the interaction of the inhibitory region of human cardiac troponin I with the C-domain of human cardiac troponin C. Biochemistry 41, 7267–7274 10.1021/bi020100c 12044157

[B100] Liu B. Lee R. S. Biesiadecki B. J. Tikunova S. B. Davis J. P. (2012). Engineered Troponin C constructs correct disease-related cardiac myofilament calcium sensitivity. J. Biol. Chem. 287, 20027–20036 10.1074/jbc.M111.334953 22511780 PMC3370186

[B101] Liu R. Feng H. Z. Jin J. P. (2014). Physiological contractility of cardiomyocytes in the wall of mouse and rat azygos vein. Am. J. Physiol. Cell Physiol. 306, C697–C704 10.1152/ajpcell.00004.2014 24477237 PMC3962596

[B102] Lu Q. W. Hinken A. C. Patrick S. E. Solaro R. J. Kobayashi T. (2010). Phosphorylation of cardiac troponin I at protein kinase C site threonine 144 depresses cooperative activation of thin filaments. J. Biol. Chem. 285, 11810–11817 10.1074/jbc.M109.055657 20164197 PMC2852917

[B103] Lüss H. Meissner A. Rolf N. Van Aken H. Bokník P. Kirchhefer U. (2000). Biochemical mechanism (s) of stunning in conscious dogs. Am. J. Physiol. Heart Circ. Physiol. 279, H176–H184 10899054 10.1152/ajpheart.2000.279.1.H176

[B104] Macgowan G. A. Du C. Cowan D. B. Stamm C. McGowan F. X. Solaro R. J. (2001). Ischemic dysfunction in transgenic mice expressing troponin I lacking protein kinase C phosphorylation sites. Am. J. Physiol. Heart Circ. Physiol. 280, H835–H843 11158984 10.1152/ajpheart.2001.280.2.H835

[B105] Maron B. J. Shirani J. Poliac L. C. Mathenge R. Roberts W. C. Mueller F. O. (1996). Sudden death in young competitive athletes. JAMA 276, 199–204 10.1001/jama.1996.03540030033028 8667563

[B106] Martin A. F. (1981). Turnover of cardiac troponin subunits. Kinetic evidence for a precursor pool of troponin-I. J. Biol. Chem. 256, 964–968 7451483

[B107] McConnell B. K. Moravec C. S. Bond M. (1998). Troponin I phosphorylation and myofilament calcium sensitivity during decompensated cardiac hypertrophy. Am. J. Physiol. Heart Circ. Physiol. 274, H385–H396 9486239 10.1152/ajpheart.1998.274.2.H385

[B108] McDonough J. L. Arrell D. K. Van Eyk J. E. (1999). Troponin I degradation and covalent complex formation accompanies myocardial ischemia/reperfusion injury. Circ. Res. 84, 9–20 10.1161/01.RES.84.1.9 9915770

[B109] McDonough J. Labugger R. Pickett W. Tse M. Mackenzie S. Pang S. (2001). Cardiac troponin I is modified in the myocardium of bypass patients. Circulation 103, 58–64 10.1161/01.CIR.103.1.58 11136686

[B110] Mesnard L. Logeart D. Taviaux S. Diriong S. Mercadier J. J. Samson F. (1995). Human cardiac troponin T: cloning and expression of new isoforms in the normal and failing heart. Circ. Res. 76, 687–692 10.1161/01.RES.76.4.687 7895342

[B111] Messer A. E. Jacques A. M. Marston S. B. (2007). Troponin phosphorylation and regulatory function in human heart muscle: dephosphorylation of Ser23/24 on troponin I could account for the contractile defect in end-stage heart failure. J. Mol. Cell. Cardiol. 42, 247–259 10.1016/j.yjmcc.2006.08.017 17081561

[B112] Mirza M. Marston S. Willott R. Ashley C. Mogensen J. McKenna W. (2005). Dilated cardiomyopathy mutations in three thin filament regulatory proteins result in a common functional phenotype. J. Biol. Chem. 280, 28498–28506 10.1074/jbc.M412281200 15923195

[B113] Mogensen J. Murphy R. T. Shaw T. Bahl A. Redwood C. Watkins H. (2004). Severe disease expression of cardiac troponin C and T mutations in patients with idiopathic dilated cardiomyopathy. J. Am. Coll. Cardiol. 44, 2033–2040 10.1016/j.jacc.2004.08.027 15542288

[B114] Monasky M. M. Taglieri D. M. Patel B. G. Chernoff J. Wolska B. M. Ke Y. (2012). p21-activated kinase improves cardiac contractility during ischemia-reperfusion concomitant with changes in troponin-T and myosin light chain 2 phosphorylation. Am. J. Physiol. Heart Circ. Physiol. 302, H224–H230 10.1152/ajpheart.00612.2011 22037191 PMC3334232

[B115] Montgomery D. E. Chandra M. Huang Q. Jin J. Solaro R. J. (2001). Transgenic incorporation of skeletal TnT into cardiac myofilaments blunts PKC-mediated depression of force. Am. J. Physiol. Heart Circ. Physiol. 280, H1011–H1018 11179042 10.1152/ajpheart.2001.280.3.H1011

[B116] Morris E. P. Lehrer S. S. (1984). Troponin-tropomyosin interactions. Fluorescence studies of the binding of troponin, troponin T and chymotryptic troponin T fragments to specifically labeled tropomyosin. Biochemistry 23, 2214–2220 10.1021/bi00305a018 6733084

[B117] Murphy A. M. (2006). Heart failure, myocardial stunning, and troponin: a key regulator of the cardiac myofilament. Congest Heart Fail 12, 32–40 10.1111/j.1527-5299.2006.04320.x 16470090

[B118] Murphy A. M. Kögler H. Georgakopoulos D. McDonough J. L. Kass D. A. Van Eyk J. E. (2000). Transgenic mouse model of stunned myocardium. Science 287, 488–491 10.1126/science.287.5452.488 10642551

[B119] Narolska N. A. Piroddi N. Belus A. Boontje N. M. Scellini B. Deppermann S. (2006). Impaired diastolic function after exchange of endogenous troponin I with C-terminal truncated troponin I in human cardiac muscle. Circ. Res. 99, 1012–1020 10.1161/01.RES.0000248753.30340.af 17023673

[B120] Nixon B. R. Thawornkaiwong A. Jin J. Brundage E. A. Little S. C. Davis J. P. (2012). AMP-activated protein kinase phosphorylates cardiac Troponin I at Ser-150 to increase myofilament calcium sensitivity and blunt PKA-dependent function. J. Biol. Chem. 287, 19136–19147 10.1074/jbc.M111.323048 22493448 PMC3365946

[B121] Noland T. A. Jr. Kuo J. F. (1991). Protein kinase C phosphorylation of cardiac troponin I or troponin T inhibits Ca^2+^-stimulated actomyosin MgATPase activity. J. Biol. Chem. 266, 4974–4978 1825828

[B122] Noland T. A. Jr. Kuo J. F. (1993). Protein kinase C phosphorylation of cardiac troponin I and troponin T inhibits Ca^2+^-stimulated MgATPase activity in reconstituted actomyosin and isolated myofibrils, and decreases actin-myosin interactions. J. Mol. Cell. Cardiol. 25, 53–65 10.1006/jmcc.1993.1007 8441181

[B123] Ogut O. Jin J. P. (1996). Expression, zinc-affinity purification, and characterization of a novel metal-binding cluster in troponin T: metal-stabilized alpha-helical structure and effects of the NH2-terminal variable region on the conformation of intact troponin T and its association with tropomyosin. Biochemistry 35, 16581–16590 10.1021/bi961712y 8987993

[B124] Oliveira S. M. Zhang Y. H. Solis R. S. Isackson H. Bellahcene M. Yavari A. (2012). AMP-activated protein kinase phosphorylates cardiac Troponin I and alters contractility of murine ventricular MyocytesNovelty and significance. Circ. Res. 110, 1192–1201 10.1161/CIRCRESAHA.111.259952 22456184

[B125] Palpant N. J. Houang E. M. Delport W. Hastings K. E. M. Onufriev A. V. Sham Y. Y. (2010). Pathogenic peptide deviations support a model of adaptive evolution of chordate cardiac performance by troponin mutations. Physiol. Genomics 42, 287–299 10.1152/physiolgenomics.00033.2010 20423961 PMC3032286

[B126] Parvatiyar M. S. Landstrom A. P. Figueiredo-Freitas C. Potter J. D. Ackerman M. J. Pinto J. R. (2012). A mutation in TNNC1-encoded cardiac troponin C, TNNC1-A31S, predisposes to hypertrophic cardiomyopathy and ventricular fibrillation. J. Biol. Chem. 287, 31845–31855 10.1074/jbc.M112.377713 22815480 PMC3442518

[B127] Perry S. (1998). Troponin T: genetics, properties and function. J. Muscle Res. Cell Motil. 19, 575–602 10.1023/A:1005397501968 9742444

[B128] Perry S. (1999). Troponin I: inhibitor or facilitator. Mol. Cell. Biochem. 190, 9–32 10.1023/A:1006939307715 10098965

[B129] Pfleiderer P. Sumandea M. P. Rybin V. O. Wang C. Steinberg S. F. (2009). Raf-1: a novel cardiac troponin T kinase. J. Muscle Res. Cell Motil. 30, 67–72 10.1007/s10974-009-9176-y 19381846 PMC2893395

[B130] Pi Y. Q. Kemnitz K. R. Zhang D. Kranias E. G. Walker J. W. (2002). Phosphorylation of troponin I controls cardiac twitch dynamics evidence from phosphorylation site mutants expressed on a troponin I-null background in mice. Circ. Res. 90, 649–656 10.1161/01.RES.0000014080.82861.5F 11934831

[B131] Pi Y. Q. Zhang D. Kemnitz K. R. Wang H. Walker J. W. (2003). Protein kinase C and A sites on troponin I regulate myofilament Ca^2+^ sensitivity and ATPase activity in the mouse myocardium. J. Physiol. 552, 845–857 10.1113/jphysiol.2003.045260 12923217 PMC2343448

[B132] Pinto J. R. Parvatiyar M. S. Jones M. A. Liang J. Ackerman M. J. Potter J. D. (2009). A functional and structural study of Troponin C mutations related to hypertrophic cardiomyopathy. J. Biol. Chem. 284, 19090–19100 10.1074/jbc.M109.007021 19439414 PMC2707221

[B133] Prigozy T. I. Dalrymple K. Shuler C. Kedes L. (1997). Differential expression of troponin C genes during tongue myogenesis. Dev. Dyn. 209, 36–44 9142494 10.1002/(SICI)1097-0177(199705)209:1<36::AID-AJA4>3.0.CO;2-Y

[B134] Quirk P. G. Patchell V. B. Gao Y. Levine B. A. Victor Perry S. (1995). Sequential phosphorylation of adjacent serine residues on the N-terminal region of cardiac troponin-I: Structure-activity implications of ordered phosphorylation. FEBS Lett. 370, 175–178 10.1016/0014-5793(95)00812-N 7656971

[B135] Rao V. S. Korte F. S. Razumova M. V. Feest E. R. Hsu H. Irving T. C. (2012). N-terminal phosphorylation of cardiac troponin-I reduces length dependent calcium sensitivity of contraction in cardiac muscle. J. Physiol. 591, 475–490 10.1113/jphysiol.2012.241604 23129792 PMC3577517

[B136] Ricchiuti V. Apple F. S. (1999). RNA expression of cardiac troponin T isoforms in diseased human skeletal muscle. Clin. Chem. 45, 2129–2135 10585344

[B137] Robertson S. Johnson J. D. Potter J. (1981). The time-course of Ca^2+^ exchange with calmodulin, troponin, parvalbumin, and myosin in response to transient increases in Ca^2+^. Biophys. J. 34, 559–569 10.1016/S0006-3495(81)84868-0 7195747 PMC1327493

[B138] Saggin L. Gorza L. Ausoni S. Schiaffino S. (1989). Troponin I switching in the developing heart. J. Biol. Chem. 264, 16299–16302 2777792

[B139] Sakthivel S. Finley N. L. Rosevear P. R. Lorenz J. N. Gulick J. Kim S. (2005). *In vivo* and *in vitro* analysis of cardiac troponin I phosphorylation. J. Biol. Chem. 280, 703–714 10.1074/jbc.M409513200 15507454

[B140] Sancho Solis R. Ge Y. Walker J. W. (2008). Single amino acid sequence polymorphisms in rat cardiac troponin revealed by top–down tandem mass spectrometry. J. Muscle Res. Cell Motil. 29, 203–212 10.1007/s10974-009-9168-y 19165611 PMC3312389

[B141] Sancho Solis R. Ge Y. Walker J. W. (2011). A preferred AMPK phosphorylation site adjacent to the inhibitory loop of cardiac and skeletal troponin I. Protein Sci. 20, 894–907 10.1002/pro.623 21416543 PMC3125873

[B142] Sasse S. Brand N. Kyprianou P. Dhoot G. Wade R. Arai M. (1993). Troponin I gene expression during human cardiac development and in end-stage heart failure. Circ. Res. 72, 932–938 10.1161/01.RES.72.5.932 8477526

[B143] Schaertl S. Lehrer S. Geeves M. (1995). Separation and characterization of the two functional regions of troponin involved in muscle thin filament regulation. Biochemistry 34, 15890–15894 10.1021/bi00049a003 8519745

[B144] Schmidtmann A. Lindow C. Villard S. Heuser A. Mügge A. Geßner R. (2005). Cardiac troponin C-L29Q, related to hypertrophic cardiomyopathy, hinders the transduction of the protein kinase A dependent phosphorylation signal from cardiac troponin I to C. FEBS J. 272, 6087–6097 10.1111/j.1742-4658.2005.05001.x 16302972

[B145] Schreier T. Kedes L. Gahlmann R. (1990). Cloning, structural analysis, and expression of the human slow twitch skeletal muscle/cardiac troponin C gene. J. Biol. Chem. 265, 21247–21253 2250022

[B146] Sehnert A. J. Huq A. Weinstein B. M. Walker C. Fishman M. Stainier D. Y. R. (2002). Cardiac troponin T is essential in sarcomere assembly and cardiac contractility. Nat. Genet. 31, 106–110 10.1038/ng875 11967535

[B147] Seidman J. G. Seidman C. (2001). The genetic basis for cardiomyopathy: from mutation identification to mechanistic paradigms. Cell 104, 557–567 10.1016/S0092-8674(01)00242-2 11239412

[B148] Sfichi-Duke L. Garcia-Cazarin M. L. Sumandea C. A. Sievert G. A. Balke C. W. Zhan D. Y. (2010). Cardiomyopathy-causing deletion K210 in cardiac troponin T alters phosphorylation propensity of sarcomeric proteins. J. Mol. Cell. Cardiol. 48, 934–942 10.1016/j.yjmcc.2010.01.005 20079745 PMC2854196

[B149] Sheng Z. Strauss W. L. Francois J. M. Potter J. D. (1990). Evidence that both Ca^2+^-specific sites of skeletal muscle TnC are required for full activity. J. Biol. Chem. 265, 21554–21560 2254314

[B150] Sherman A. J. Klocke F. J. Decker R. S. Decker M. L. Kozlowski K. A. Harris K. R. (2000). Myofibrillar disruption in hypocontractile myocardium showing perfusion-contraction matches and mismatches. Am. J. Physiol. Heart Circ. Physiol. 278, H1320–H1334 10749730 10.1152/ajpheart.2000.278.4.H1320

[B151] Solaro R. J. (2010). Sarcomere control mechanisms and the dynamics of the cardiac cycle. J. Biomed. Biotechnol. 2010:105648 10.1155/2010/105648 20467475 PMC2866969

[B152] Solaro R. J. Kobayashi T. (2011). Protein phosphorylation and signal transduction in cardiac thin filaments. J. Biol. Chem. 286, 9935–9940 10.1074/jbc.R110.197731 21257760 PMC3060547

[B153] Solaro R. J. Lee J. A. Kentish J. C. Allen D. G. (1988). Effects of acidosis on ventricular muscle from adult and neonatal rats. Circ. Res. 63, 779–787 10.1161/01.RES.63.4.779 3168178

[B154] Solaro R. J. Rosevear P. Kobayashi T. (2008). The unique functions of cardiac troponin I in the control of cardiac muscle contraction and relaxation. Biochem. Biophys. Res. Commun. 369, 82–87 10.1016/j.bbrc.2007.12.114 18162178 PMC2365727

[B155] Solaro R. J. Van Der Velden J. (2010). Why does troponin I have so many phosphorylation sites? Fact and fancy. J. Mol. Cell. Cardiol. 48, 810–816 10.1016/j.yjmcc.2010.02.014 20188739 PMC2854207

[B156] Stelzer J. E. Patel J. R. Walker J. W. Moss R. L. (2007). Differential roles of cardiac myosin-binding protein C and cardiac troponin I in the myofibrillar force responses to protein kinase A phosphorylation. Circ. Res. 101, 503–511 10.1161/CIRCRESAHA.107.153650 17641226

[B157] Stevens L. Bastide B. Kischel P. Pette D. Mounier Y. (2002). Time-dependent changes in expression of troponin subunit isoforms in unloaded rat soleus muscle. Am. J. Physiol. Cell Physiol. 282, C1025–C1030 10.1152/ajpcell.00252.2001 11940518

[B158] Sumandea M. P. Pyle W. G. Kobayashi T. De Tombe P. P. Solaro R. J. (2003). Identification of a functionally critical protein kinase C phosphorylation residue of cardiac troponin T. J. Biol. Chem. 278, 35135–35144 10.1074/jbc.M306325200 12832403

[B159] Sumandea M. P. Vahebi S. Sumandea C. A. Garcia-Cazarin M. L. Staidle J. Homsher E. (2009). Impact of cardiac troponin T N-terminal deletion and phosphorylation on myofilament function. Biochemistry 48, 7722–7731 10.1021/bi900516n 19586048

[B160] Sweeney H. L. Brito R. Rosevear P. R. Putkey J. A. (1990). The low-affinity Ca^2+^-binding sites in cardiac/slow skeletal muscle troponin C perform distinct functions: site I alone cannot trigger contraction. Proc. Natl. Acad. Sci. U.S.A. 87, 9538–9542 10.1073/pnas.87.24.9538 2263608 PMC55207

[B161] Taglieri D. M. Monasky M. M. Knezevic I. Sheehan K. A. Lei M. Wang X. (2011). Ablation of p21-activated kinase-1 in mice promotes isoproterenol-induced cardiac hypertrophy in association with activation of Erk1/2 and inhibition of protein phosphatase 2A. J. Mol. Cell. Cardiol. 51, 988–996 10.1016/j.yjmcc.2011.09.016 21971074 PMC3208757

[B162] Takeda S. Yamashita A. Maeda K. Maeda Y. (2003). Structure of the core domain of human cardiac troponin in the Ca^2+^-saturated form. Nature 424, 35–41 10.1038/nature01780 12840750

[B163] Tanokura M. Tawada Y. Ono A. Ohtsuki I. (1983). Chymotryptic subfragments of troponin T from rabbit skeletal muscle. Interaction with tropomyosin, troponin I and troponin C. J. Biochem. (Tokyo) 93, 331–337 6841341 10.1093/oxfordjournals.jbchem.a134185

[B164] Tardiff J. C. Factor S. M. Tompkins B. D. Hewett T. E. Palmer B. M. Moore R. L. (1998). A truncated cardiac troponin T molecule in transgenic mice suggests multiple cellular mechanisms for familial hypertrophic cardiomyopathy. J. Clin. Invest. 101, 2800 10.1172/JCI2389 9637714 PMC508871

[B165] Thierfelder L. Watkins H. Macrae C. Lamas R. McKenna W. Vosberg H.-P. (1994). α-tropomyosin and cardiac troponin T mutations cause familial hypertrophic cardiomyopathy: a disease of the sarcomere. Cell 77, 701–712 10.1016/0092-8674(94)90054-X 8205619

[B166] Thomas S. A. Fallavollita J. A. Lee T. C. Feng J. Canty J. M. (1999). Absence of troponin I degradation or altered sarcoplasmic reticulum uptake protein expression after reversible ischemia in swine. Circ. Res. 85, 446–456 10.1161/01.RES.85.5.446 10473674

[B167] Townsend P. J. Barton P. J. Yacoub M. H. Farza H. (1995). Molecular cloning of human cardiac troponin T isoforms: expression in developing and failing heart. J. Mol. Cell. Cardiol. 27, 2223–2236 10.1016/S0022-2828(95)91587-7 8576938

[B168] Vahebi S. Kobayashi T. Warren C. M. De Tombe P. P. Solaro R. J. (2005). Functional effects of rho-kinase–dependent phosphorylation of specific sites on cardiac troponin. Circ. Res. 96, 740–747 10.1161/01.RES.0000162457.56568.7d 15774859

[B169] Van Der Velden J. Merkus D. Klarenbeek B. James A. Boontje N. Dekkers D. (2004). Alterations in myofilament function contribute to left ventricular dysfunction in pigs early after myocardial infarction. Circ. Res. 95, e85–e95 10.1161/01.RES.0000149531.02904.09 15528471

[B170] Van Eerd J. P. Takahashi K. (1976). Determination of the complete amino acid sequence of bovine cardiac troponin C. Biochemistry 15, 1171–1180 10.1021/bi00650a033 1252434

[B171] Varnava A. M. Elliott P. M. Baboonian C. Davison F. Davies M. J. McKenna W. J. (2001). Hypertrophic cardiomyopathy histopathological features of sudden death in cardiac Troponin T disease. Circulation 104, 1380–1384 10.1161/hc3701.095952 11560853

[B172] Vinogradova M. V. Stone D. B. Malanina G. G. Karatzaferi C. Cooke R. Mendelson R. A. (2005). Ca^2+^-regulated structural changes in troponin. Proc. Natl. Acad. Sci. U.S.A. 102, 5038–5043 10.1073/pnas.0408882102 15784741 PMC555973

[B173] Wang D. Robertson I. M. Li M. X. McCully M. E. Crane M. L. Luo Z. (2012a). Structural and functional consequences of the cardiac Troponin C L48Q Ca^2+^-sensitizing mutation. Biochemistry 51, 4473–4487 10.1021/bi3003007 22591429 PMC3437384

[B174] Wang H. Grant J. E. Doede C. M. Sadayappan S. Robbins J. Walker J. W. (2006). PKC-betaII sensitizes cardiac myofilaments to Ca^2+^ by phosphorylating troponin I on threonine-144. J. Mol. Cell. Cardiol. 41, 823 10.1016/j.yjmcc.2006.08.016 17010989

[B175] Wang J. Jin J. P. (1998). Conformational modulation of troponin T by configuration of the NH2-terminal variable region and functional effects. Biochemistry 37, 14519–14528 10.1021/bi9812322 9772180

[B176] Wang X. Huang Q. Q. Breckenridge M. T. Chen A. Crawford T. O. Morton D. H. (2005). Cellular fate of truncated slow skeletal muscle troponin T produced by Glu180 nonsense mutation in amish nemaline myopathy. J. Biol. Chem. 280, 13241–13249 10.1074/jbc.M413696200 15665378

[B177] Wang Y. Pinto J. R. Solis R. S. Dweck D. Liang J. Diaz-Perez Z. (2012b). Generation and functional characterization of knock-in mice harboring the cardiac Troponin I-R21C mutation associated with hypertrophic cardiomyopathy. J. Biol. Chem. 287, 2156–2167 10.1074/jbc.M111.294306 22086914 PMC3265894

[B178] Ward A. J. Cooper T. A. (2010). The pathobiology of splicing. J. Pathol. 220, 152–163 10.1002/path.2649 19918805 PMC2855871

[B179] Watkins H. McKenna W. J. Thierfelder L. Suk H. J. Anan R. O'donoghue A. (1995). Mutations in the genes for cardiac troponin T and α-tropomyosin in hypertrophic cardiomyopathy. N. Engl. J. Med. 332, 1058–1065 7898523 10.1056/NEJM199504203321603

[B180] Wei B. Gao J. Huang X. P. Jin J. P. (2010). Mutual rescues between two dominant negative mutations in cardiac troponin I and cardiac troponin T. J. Biol. Chem. 285, 27806–27816 10.1074/jbc.M110.137844 20551314 PMC2934648

[B181] Wei B. Jin J. P. (2011). Troponin T isoforms and posttranscriptional modifications: evolution, regulation and function. Arch. Biochem. Biophys. 505, 144–154 10.1016/j.abb.2010.10.013 20965144 PMC3018564

[B182] Wei H. Jin J. P. (2013). N-terminal truncated cardiac troponin I enhanced the contractility of isolated cardiomyocytes. Biophys. J. 104, 154a–155a 10.1016/j.bpj.2012.11.875

[B183] Westfall M. V. Albayya F. P. Metzger J. M. (1999). Functional analysis of troponin I regulatory domains in the intact myofilament of adult single cardiac myocytes. J. Biol. Chem. 274, 22508–22516 10.1074/jbc.274.32.22508 10428827

[B184] Westfall M. V. Albayya F. P. Turner I. I. Metzger J. M. (2000). Chimera analysis of troponin I domains that influence Ca^2+^-activated myofilament tension in adult cardiac myocytes. Circ. Res. 86, 470–477 10.1161/01.RES.86.4.470 10700453

[B185] Westfall M. V. Lee A. M. Robinson D. A. (2005). Differential contribution of Troponin I phosphorylation sites to the endothelin-modulated contractile response. J. Biol. Chem. 280, 41324–41331 10.1074/jbc.M506043200 16236710

[B186] White S. P. Cohen C. Phillips G. N. Jr. (1987). Structure of co-crystals of tropomyosin and troponin. Nature 325, 826–828 10.1038/325826a0 3102969

[B187] Willott R. H. Gomes A. V. Chang A. N. Parvatiyar M. S. Pinto J. R. Potter J. D. (2010). Mutations in Troponin that cause HCM, DCM AND RCM: What can we learn about thin filament function? J. Mol. Cell. Cardiol. 48, 882–892 10.1016/j.yjmcc.2009.10.031 19914256

[B188] You B. Yan G. Zhang Z. Yan L. Li J. Ge Q. (2009). Phosphorylation of cardiac troponin I by mammalian sterile 20-like kinase 1. Biochem. J. 418, 93–101 10.1042/BJ20081340 18986304 PMC2754779

[B189] Yu Z. B. Gao F. Feng H. Z. Jin J. P. (2007). Differential regulation of myofilament protein isoforms underlying the contractility changes in skeletal muscle unloading. Am. J. Physiol. Cell Physiol. 292, C1192–C1203 10.1152/ajpcell.00462.2006 17108008 PMC1820608

[B190] Yu Z. B. Wei H. Jin J. P. (2012). Chronic coexistence of two troponin T isoforms in adult transgenic mouse cardiomyocytes decreased contractile kinetics and caused dilatative remodeling. Am. J. Physiol. Cell Physiol. 303, C24–C32 10.1152/ajpcell.00026.2012 22538236 PMC3404524

[B191] Yu Z. B. Zhang L. F. Jin J. P. (2001). A proteolytic NH2-terminal truncation of cardiac troponin I that is up-regulated in simulated microgravity. J. Biol. Chem. 276, 15753–15760 10.1074/jbc.M011048200 11278823

[B192] Yumoto F. Lu Q. W. Morimoto S. Tanaka H. Kono N. Nagata K. (2005). Drastic Ca^2+^ sensitization of myofilament associated with a small structural change in troponin I in inherited restrictive cardiomyopathy. Biochem. Biophys. Res. Commun. 338, 1519–1526 10.1016/j.bbrc.2005.10.116 16288990

[B193] Zakhary D. R. Moravec C. S. Stewart R. W. Bond M. (1999). Protein kinase A (PKA)-dependent troponin-I phosphorylation and PKA regulatory subunits are decreased in human dilated cardiomyopathy. Circulation 99, 505–510 10.1161/01.CIR.99.4.505 9927396

[B194] Zhang P. Kirk J. A. Ji W. Dos Remedios C. G. Kass D. A. Van Eyk J. E. (2012). Multiple reaction monitoring to identify site-specific Troponin I phosphorylated residues in the failing human heart/clinical perspective. Circulation 126, 1828–1837 10.1161/CIRCULATIONAHA.112.096388 22972900 PMC3733556

[B195] Zhang R. Zhao J. Mandveno A. Potter J. D. (1995a). Cardiac troponin I phosphorylation increases the rate of cardiac muscle relaxation. Circ. Res. 76, 1028–1035 10.1161/01.RES.76.6.1028 7758157

[B196] Zhang R. Zhao J. J. Potter J. D. (1995b). Phosphorylation of both serine residues in cardiac troponin I is required to decrease the Ca affinity of cardiac troponin C. J. Biol. Chem. 270, 30773–30780 10.1074/jbc.270.51.30773 8530519

[B197] Zhang Z. Akhter S. Mottl S. Jin J. P. (2011a). Calcium-regulated conformational change in the C-terminal end segment of troponin I and its binding to tropomyosin. FEBS J. 278, 3348–3359 10.1111/j.1742-4658.2011.08250.x 21777381 PMC3168705

[B198] Zhang Z. Biesiadecki B. J. Jin J. P. (2006). Selective deletion of the NH2-terminal variable region of cardiac troponin T in ischemia reperfusion by myofibril-associated mu-calpain cleavage. Biochemistry 45, 11681–11694 10.1021/bi060273s 16981728 PMC1762003

[B199] Zhang Z. Feng H. Z. Jin J. P. (2011b). Structure of the NH2-terminal variable region of cardiac troponin T determines its sensitivity to restrictive cleavage in pathophysiological adaptation. Arch. Biochem. Biophys. 515, 37–45 10.1016/j.abb.2011.08.013 21924234 PMC3192527

[B200] Zot H. Potter J. (1982). A structural role for the Ca^2+^-Mg^2+^ sites on troponin C in the regulation of muscle contraction. Preparation and properties of troponin C depleted myofibrils. J. Biol. Chem. 257, 7678–7683 6211445

